# Quantification in wavelength-resolved neutron imaging: exemplified using polyethylene to mimic hydrogen in stainless steel

**DOI:** 10.1107/S2052252526008882

**Published:** 2026-10-22

**Authors:** Shuqi Xu, Thawatchart Chulapakorn, Carl F. O. Dahlberg, Jose Ignacio Marquez Damian, Menghao Liu, Douglas D. DiJulio, Thomas Kittelmann, Adrian Losko, Stefanos Athanasopoulos, Jinshan Pan, Alfred Larsson, Gunter Muhrer, Winfried Kockelmann, Ranggi S. Ramadhan, Alexander Wolfertz, Robin Woracek

**Affiliations:** a European Spallation Source ERIC Partikelgatan 5 SE-224 84 Lund Sweden; b Division of Synchrotron Radiation Research, Department of Physics, Lund University, Box 118, SE-221 00 Lund, Sweden; c Division of Surface and Corrosion Science, Department of Chemistry, KTH Royal Institute of Technology, Drottning Kristinas väg. 51, SE-100 44 Stockholm, Sweden; d Department of Engineering Mechanics, KTH Royal Institute of Technology, Teknikringen 8D, SE-100 44 Stockholm, Sweden; e ESS Data Management and Scientific Computing Division, Lyngby, Denmark; f Forschungs-Neutronenquelle Heinz Maier-Leibnitz, 85748 Garching, Germany; g ISIS Facility, Rutherford Appleton Laboratory, Chilton, Didcot OX11 0QX, UK; Tsinghua University, China

**Keywords:** materials science, computational modelling, neutron imaging, hydrogen quantification, hydrogen detection limit

## Abstract

We present a systematic and improved quantitative methodology for analysing wavelength-resolved neutron transmission spectra in metallic alloys. The approach is demonstrated using data acquired on both the IMAT beamline at ISIS and the RADEN beamline at J-PARC on Super Duplex stainless steel samples partially covered with thin polyethylene layers to mimic small controlled hydrogen concentrations. Initially, we employed Monte Carlo particle transport simulations to assess the effect of forward-scattered neutrons on the recorded transmission signal as a function of sample-to-detector distance and neutron beam size. Guided by this simulation framework, we define an experimental configuration and data processing workflow that enables the reliable quantification of very small transmission differences, reaching the sub-percent regime. An *NCrystal*-based least-squares fitting routine is developed to resolve multiple microstructural parameters simultaneously, including unit-cell parameters, phase fractions, element composition and extinction-related parameters. The model quantitatively reproduces the experimental transmission spectra and determines polyethylene layer thicknesses with micrometre-level precision. This work establishes a robust microstructure-sensitive framework for quantitative neutron transmission analysis. The approach enables the quantification of trace elements that produce measurable contrasts in neutron transmission, exemplified here for hydrogen in steel with a demonstrated detection limit of 10 wt p.p.m. The presented workflow provides a broadly applicable framework for improving quantitative neutron imaging across diverse materials and applications.

## Abstract 2

We have developed a comprehensive quantitative methodology for analysing wavelength-resolved neutron transmission spectra and here demonstrate its application to metallic alloys. The approach integrates Monte Carlo simulations, which serve as a digital twin of the experiment, with *NCrystal*-based least-squares fitting. By using open-source software, this method resolves and quantifies trace element contributions, as demonstrated here for detecting hydrogen-equivalent concentrations below 10 wt p.p.m. in stainless steel.

## 1. Introduction

Wavelength-resolved neutron transmission imaging is a powerful nondestructive technique for characterizing the microstructural properties of materials, with diverse applications in materials science and engineering research domains, such as energy materials and cultural heritage studies (Strobl & Lehmann, 2024 ▸; Aswal *et al.*, 2022 ▸; Woracek *et al.*, 2018 ▸; Kardjilov *et al.*, 2018 ▸; Tran *et al.*, 2021 ▸). When an incident neutron beam traverses a sample, its intensity is attenuated due to nuclear interactions, primarily scattering and absorption, as governed by the Beer–Lambert law. The resulting wavelength-dependent transmission spectrum encodes detailed information about the material’s structural and compositional characteristics, including elemental com­position and crystallographic properties. For polycrystalline materials, Bragg edge analysis provides access to phase fractions, unit-cell parameters, strain states, preferred orientation (texture) and other microstructural effects that cause, for example, extinction.

This study is motivated by the need for quantitative determination of very low hydrogen concentrations in metallic materials. While the quantification of trace elements is relevant in many fields, this challenge is particularly critical in the study of hydrogen embrittlement (HE), where even trace amounts of hydrogen can lead to significant degradation of mechanical properties. HE is a complex multiscale phenomenon involving hydrogen absorption, diffusion, trapping and interactions with microstructural features, ultimately compromising the ductility, toughness and fatigue life in high-strength steels and Ni-based alloys (Yu *et al.*, 2024 ▸). Despite decades of research, the underlying failure mechanisms, such as hydrogen-enhanced decohesion (HEDE), hydrogen-enhanced localized plasticity (HELP) and hydrogen-enhanced strain-induced vacancy (HESIV), remain difficult to validate experimentally, in part because hydrogen concentrations in engineering alloys are typically very low.

While several experimental techniques exist for hydrogen characterization, quantitative and spatially resolved measurements in structural alloys at technologically relevant concentrations remain highly challenging. Conventional methods such as thermal desorption spectroscopy (TDS) or hot gas extraction (HGE) lack spatial resolution, while high-resolution techniques such as secondary ion mass spectrometry (SIMS) and atom probe tomography (APT) are limited by small sample volumes, demanding sample preparation, and challenges associated with the reliable detection and quantification of hydrogen. Neutron imaging, by contrast, has long been recognized as a promising technique due to its high penetration depth and intrinsic sensitivity to hydrogen, offering the potential for spatially resolved measurements in bulk materials. Although hydrogen possesses an exceptionally large incoherent neutron scattering cross section, extracting reliable quantitative information from transmission measurements has remained challenging. Notably, Buitrago *et al.* (2018 ▸) demonstrated the advantages of wavelength-resolved neutron transmission analysis for determining low hydrogen concentrations in Zr-based alloys (where stable hydrides form).

While it is possible to manufacture calibrated specimens of Zr-based alloys with well defined hydrogen concentrations, this is far more challenging for steels, from which hydrogen may effuse readily. In an ongoing research project, we are investigating the hydrogen embrittlement behaviour of stainless steels. Previously, we successfully examined delayed cracking in high-strength martensitic steel using *in situ* neutron radiography on the NeXT beamline at ILL (Lindblom *et al.*, 2024 ▸; Tengattini *et al.*, 2020 ▸). However, these studies relied on wavelength-integrated imaging and did not enable direct detection or quantification of hydrogen. This limitation arose because the hydrogen concentrations were below the detection sensitivity of the employed imaging configuration and/or localized within regions smaller than the achievable spatial resolution.

To address this challenge, we moved towards wavelength-resolved neutron imaging, which provides enhanced sensitivity to small transmission changes. In the present work, we establish a robust analysis workflow that enables the quantification of small hydrogen-equivalent signals from wavelength-resolved spectra. The methodology is demonstrated using Super Duplex stainless steel (SDSS) samples partially covered with thin polyethylene (PE) layers as a controlled proxy for low hydrogen content. While PE serves as a convenient validation case, the underlying workflow is broadly applicable to other systems requiring precise quantification of trace element contributions. We therefore expect that the methodology presented here will support future advances in quantitative neutron imaging at pulsed neutron sources in general (Shinohara *et al.*, 2020 ▸; Kockelmann *et al.*, 2018 ▸; Bilheux *et al.*, 2023 ▸; Strobl, 2015 ▸; Nelson *et al.*, 2018 ▸).

In addition to sample-related scattering effects, quantitative wavelength-resolved neutron imaging requires careful control of systematic effects arising from the experimental configuration. Detector-generated background is commonly assumed to remain unchanged between the open-beam and sample measurements used for transmission normalization. However, changes in the detector irradiation conditions caused by the introduction of a sample can produce wavelength-dependent systematic offsets in the normalized transmission that should be considered for highly quantitative measurements.

To address these systematic effects, our experimental strategy begins with Monte Carlo particle transport simulations to quantify the potential impact of neutron scattering processes, particularly forward scattering, on the measured transmission spectra. These simulations effectively act as a digital twin of the experiment, enabling us to assess and optimize the experimental configuration prior to measurement. Performing these simulations beforehand is an important step in designing an appropriate experimental configuration that ensures systematic effects remain sufficiently small for quantitative analysis.

Forward scattering refers to neutrons being scattered in the downstream direction. When the detector is positioned close to the sample, a fraction of these scattered neutrons still reach regions of the detector that are ‘covered’ by the sample, leading to an overestimation of the transmission signal. This effect is especially relevant in neutron imaging, where short sample-to-detector distances are commonly employed to minimize geometric blurring. The influence of forward scattering on quantitative transmission measurements has been well studied and several correction strategies have been proposed. For example, Kardjilov *et al.* (2005 ▸) developed a point-scattered function approach to correct for scattering contributions in neutron radiography. More recently, Boillat *et al.* (2018 ▸) introduced the black-body grid correction, which effectively compensates for systematic detector-related and geometry-induced artefacts – even beyond those arising solely from forward scattering. For a more comprehensive overview, we refer readers to Boillat *et al.* (2018 ▸) and the references therein.

While the impact of forward scattering has been previously investigated for iron samples using the Monte Carlo ray-tracing code *McStas* (Boin, 2011 ▸; Willendrup & Lefmann, 2020 ▸; Willendrup & Lefmann, 2021 ▸) and more comprehensively by Sato and co-workers using the *PHITS*+*RITS* framework (Sato *et al.*, 2017 ▸; Sato *et al.*, 2013 ▸; Sato *et al.*, 2011 ▸), we make use of recent advancements in open-source simulation tools. Specifically, we utilize the *NCrystal* toolkit (Cai *et al.*, 2019 ▸; Cai & Kittelmann, 2020 ▸; Kittelmann & Cai, 2021 ▸), which can serve as a scattering kernel backend for Monte Carlo codes including *OpenMC* (Romano *et al.*, 2015 ▸) and *McStas. NCrystal* provides the scattering physics of low-energy neutrons (including both coherent and incoherent, elastic and inelastic scattering for various materials; complementary physics such as magnetic neutron scattering is also supported via the plugin feature) while the particle transport code *OpenMC* handles complex geometry, multiple scattering and other neutron transport physics. Recent work has demonstrated the promising potential of the combination of *OpenMC* and *NCrystal* for quantitative neutron experiment modelling (Xu *et al.*, 2024 ▸). Consequently, we performed such simulations using *OpenMC* and *NCrystal* to account for forward-scattering effects accurately, thereby enabling improved experimental design and quantitative spectra analysis.

We have developed an analysis routine based on *NCrystal* and conducted neutron transmission experiments on both the IMAT (Kockelmann *et al.*, 2018 ▸) imaging instrument located at Target Station 2 of the ISIS facility and the RADEN (Shinohara *et al.*, 2020 ▸) imaging beamline at J-PARC. Recent years have seen growing adoption of *NCrystal* for transmission or imaging experiment analysis (Strobl *et al.*, 2025 ▸; Sebold *et al.*, 2024 ▸; Simoni *et al.*, 2023 ▸; Sumarli *et al.*, 2024 ▸; Minniti *et al.*, 2025 ▸; Hirsh *et al.*, 2025*a* ▸; Xu *et al.*, 2025 ▸), including work combining *NCrystal* with least-squares fitting to characterize extinction effects in beryllium metal (Xu *et al.*, 2025 ▸). Building upon these developments, we present an extended analysis pipeline for quantitative analysis of neutron transmission spectra.

This work is organized as follows. First, we present the theoretical background for the low-energy neutron cross sections of SDSS and PE. Next, we present the Monte Carlo simulations used to study the impact of forward scattering under different experimental configurations, which are then used to determine the optimal experimental configuration. We then describe the utilized neutron imaging experimental setup on IMAT and RADEN and the data processing methodology used to obtain the transmission spectra. Finally, we detail the *NCrystal*-based least-squares fitting procedure and demonstrate how small signals from thin PE layers can be reliably extracted, where the detection limit of hydrogen is derived, followed by a discussion of the implications for future quantitative neutron imaging.

## 2. Theoretical total neutron cross sections of SDSS and PE

The total neutron cross section σ(λ) (with λ being the neutron wavelength) is a wavelength-dependent measure of the probability of a neutron interacting with atoms in a material. For the non-fissionable materials SDSS and PE, σ(λ) is composed of the scattering cross section σ_scat_(λ) and the absorption cross section σ_abs_(λ), 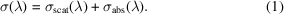
The physics of low-energy neutron scattering is detailed in the literature (Squires, 2012 ▸; Schober, 2014 ▸; Carpenter & Loong, 2015 ▸). Under the incoherent approximation, σ_scat_(λ) can be calculated by 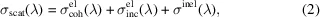
where 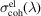, 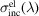 and σ^inel^(λ) represent the coherent elastic, incoherent elastic and inelastic scattering cross sections, respectively.

In this work, the open-source toolkit *NCrystal* is used to calculate the wavelength-dependent neutron scattering and absorption cross sections for SDSS and PE, taking advantage of the extensive *NCrystal* database, which includes crystalline and amorphous solids, liquids and gases. Microstructural parameters, including elemental composition, phase fractions and unit-cell parameters, are specified via NCMAT files and built-in configuration string functionalities.

**Figure 1:**
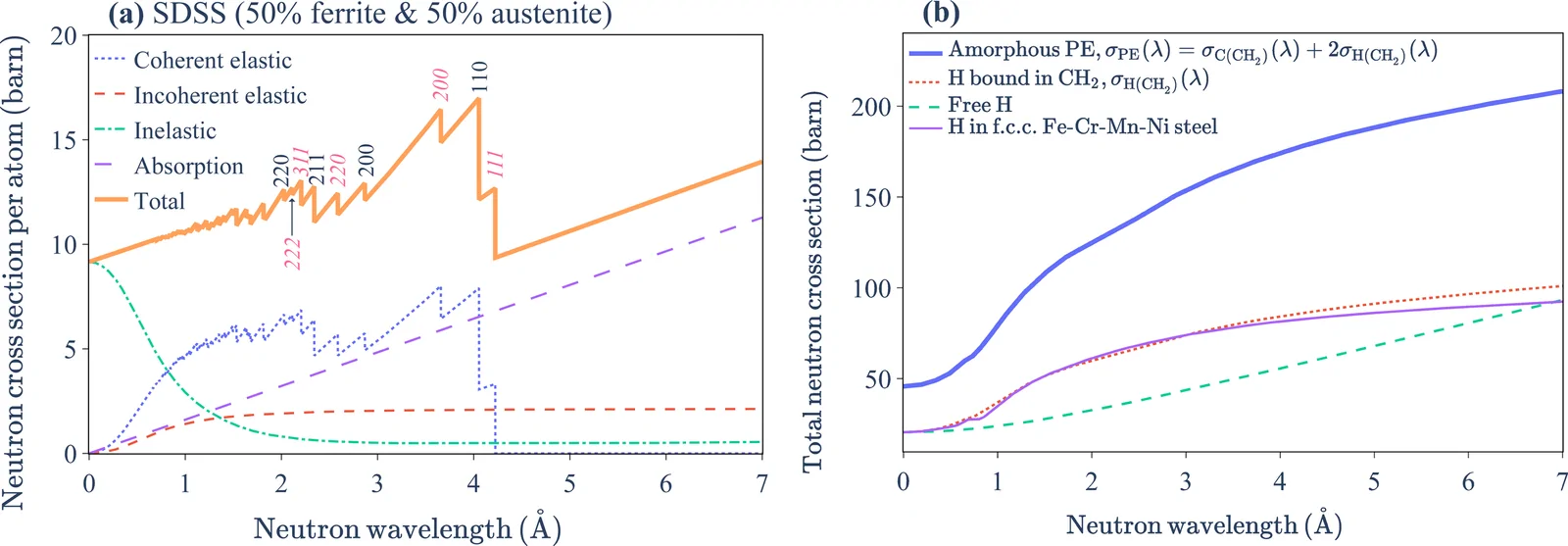
(*a*) Neutron cross sections per atom for the SDSS sample. The cross-section components defined in equations (1) and (2) are shown. Primary *hkl* reflections for both the ferrite and austenite phases are labelled, with austenite indices indicated in italics. (*b*) The total neutron cross section for amorphous PE (chemical formula CH_2_). Also plotted are the total cross sections for H bound in CH_2_, free hydrogen, and interstitial H in f.c.c. Fe–Cr–Mn–Ni steel using PDOS from Danilkin *et al.* (2003 ▸). See text for further discussion.

The material investigated in this work is Super Duplex stainless steel (SDSS) grade 2507 (UNS S32750). Its properties are detailed by AZo Materials (2025 ▸). SDSS is composed of approximately 50% ferrite (body-centred cubic, b.c.c.) and 50% austenite (face-centred cubic, f.c.c.). The nominal element compositions are summarized in Table 1 ▸. By incorporating these properties into *NCrystal*, the scattering and absorption cross sections were obtained [Fig. 1 ▸(*a*)].

It should be noted that we initially defaulted to the phonon density of states (PDOS) of pure iron in the ferrite and austenite phases to calculate the scattering cross sections of the SDSS sample. While phonon softening is a common signature in alloys compared with their pure forms, as shown, for example, by Beskrovni *et al.* (1999 ▸) and Yang *et al.* (2022 ▸), we found the effect to be negligible here. We used the PDOS of f.c.c. Fe–Cr–Ni–N austenite alloys with varying chromium contents from Beskrovni *et al.* (1999 ▸) to compute the cross sections. The resulting cross sections are nearly identical to those computed using the pure iron PDOS. Consequently, we proceeded with the pure iron PDOS for all subsequent SDSS cross-section calculations.

Fig. 1 ▸(*b*) shows the total cross section for amorphous PE (chemical formula CH_2_) σ_PE_(λ), calculated using *NCrystal*. This value is composed of the cross section of carbon bound in CH_2_, 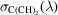, plus twice the cross section of hydrogen bound in CH_2_, 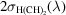. These calculations use the NCMAT file of PE available in the *NCrystal* database, which has been benchmarked against experimental data from Ramić *et al.* (2018 ▸). For comparison, the total cross sections for H bound in CH_2_ and free H are also plotted. These differences originate from the chemical bonding and dynamic state of hydrogen in the material, on one hand highlighting the importance of accounting for hydrogen dynamics for accurate quantification, and on the other hand opening new avenues for experimental investigations. Although the dynamics of hydrogen bound in PE cannot be directly used to model those in stainless steels, the workflow presented hereafter remains valid, provided the hydrogen dynamics are determined from complementary studies, such as vibrational spectroscopy measurements, density functional theory (DFT) calculations or molecular dynamics (MD) simulations.

In the present case, we investigate a Super Duplex stainless steel consisting of approximately equal fractions of ferrite (b.c.c. form) and austenite (f.c.c. form). Previous studies have shown that hydrogen is primarily trapped in austenite for ferrite–austenite dual-phase materials (Silverstein *et al.*, 2017 ▸; Zou *et al.*, 2026 ▸). Danilkin *et al.* (2003 ▸) conducted neutron vibrational spectroscopy measurements on two f.c.c. Fe-based austenite alloys doped with hydrogen. Based on their study, we extracted the PDOS of hydrogen and used it to compute the neutron total cross section. The resulting cross section shows a noticeable difference compared with that of free hydrogen [Fig. 1 ▸(*b*)]. These results demonstrate the sensitivity of the neutron signal to hydrogen dynamics, providing a framework that can be readily applied to quantify hydrogen concentrations in Fe-based alloys.

## 3. Monte Carlo simulations

Since it is well known that forward-scattered neutrons can introduce systematic artefacts in transmission measurements, we first evaluated the influence of the experimental geometry using Monte Carlo particle transport simulations, which serve as a digital twin of the experimental setup. A comprehensive investigation of such effects was conducted by Sato *et al.* (2017 ▸), who studied how parameters such as sample-to-detector distance, detection area, detection position and sample thickness influence transmission spectra. In their work, the Monte Carlo particle transport code *PHITS* was used to obtain simulated transmission spectra, while the *RITS* code was employed to compute the corresponding theoretical transmission intensities for comparison.

**Figure 2:**
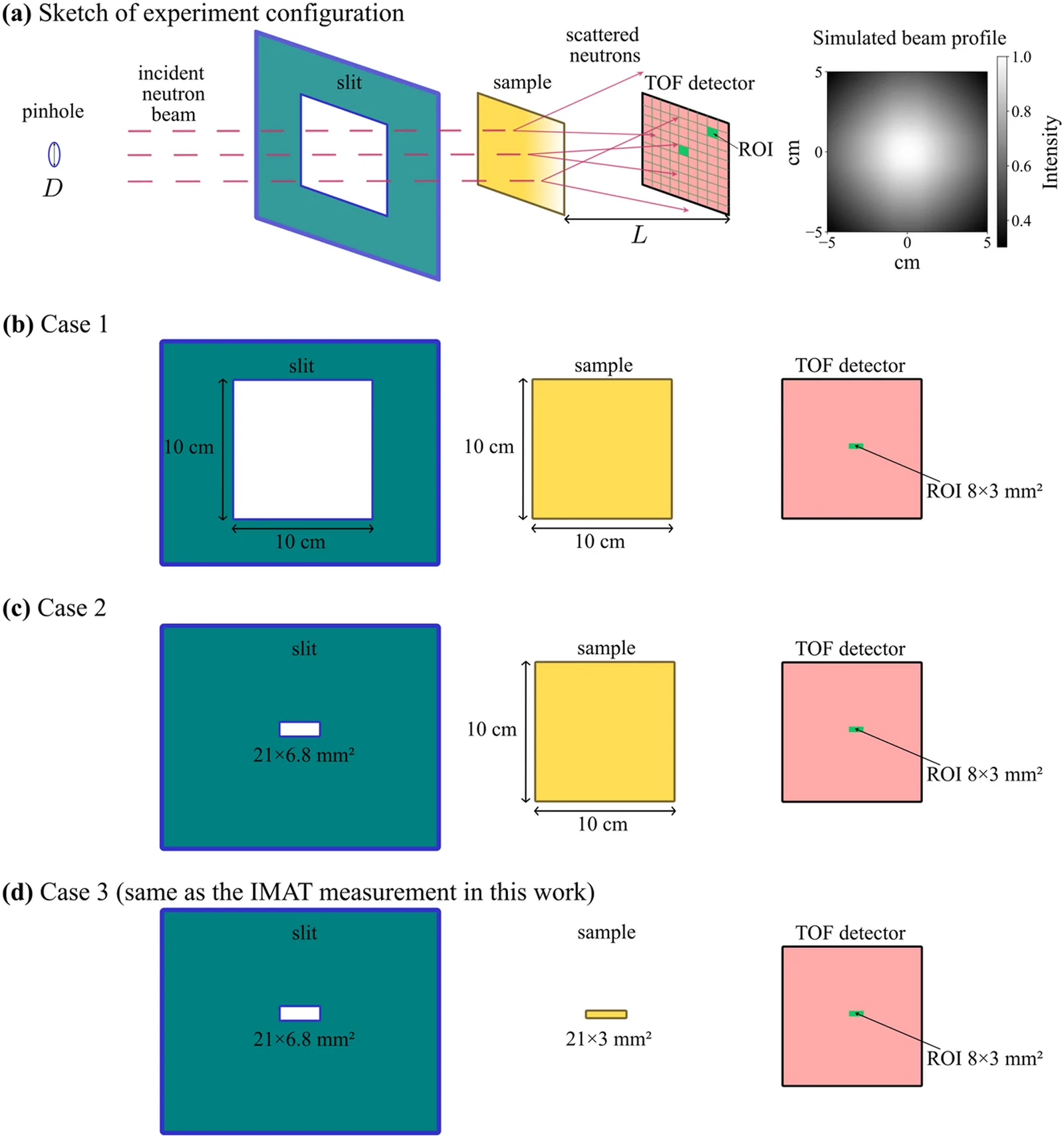
(*a*) Schematic presentation of the neutron transmission experiment configuration in *OpenMC*+*NCrystal* simulations. The incident neutron distribution follows the open-beam spectrum of IMAT, with beam divergence determined by the pinhole diameter and the distance between the pinhole and the sample. The incident neutron wavelength/energy distribution, beam divergence, sample geometry, region of interest (ROI) or detector region and sample-to-detector distance (*L*) can be flexibly adjusted in the simulations. (*b*) and (*c*) Two simulation cases where the sample dimensions are both 10 × 10 × 0.8 cm (width × height × thickness) and the ROIs are both 8 × 3 mm, but with different slit sizes. (*d*) The setup adopted for the IMAT experiment. See text for more details.

In the present study, we focus specifically on the impact of the sample-to-detector distance and the slit/beam size on the transmission spectrum using *OpenMC*+*NCrystal* simulations. The sample-to-detector distance is defined as the distance from the end of the sample to the detector sensor. We used the established work of Sato *et al.* (2017 ▸) as a benchmark for validating our Monte Carlo simulations, as detailed in Appendix *A*[App appa] (Fig. 12), ensuring the reliability of our calculations. We then conducted a systematic study to evaluate forward-scattering effects and their impact on the accuracy of transmission-based quantification. The investigation considered several experimental configurations, of which three representative cases (Cases 1–3) are presented here, as illustrated in Figs. 2 ▸(*b*)–2 ▸(*d*). While Case 3 was ultimately adopted for the experimental measurements on IMAT, Cases 1 and 2 illustrate the strong influence of slit size on the transmission spectrum.

The open-beam spectrum was taken from a previous experiment and used as input for the simulations. The beam divergence θ_div_ was determined from the experimental pinhole size *D*_ph_ and the pinhole-to-sample distance, *L*_p–s_ = 10 m, using the relationship 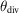 = 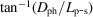 reported by Burca *et al.* (2013 ▸). With *D*_ph_ set to 60 mm – a value typically chosen to optimize the detector count rate – the resulting beam divergence was θ_div_ = 0.344°. These are key parameters set for the *OpenMC*+*NCrystal* simulations. It should be noted that neutron divergence at the guide exit is not accounted for in our simulations. In principle, more detailed beamline descriptions, using for example *McStas* or *VITESS* (Zendler *et al.*, 2014 ▸; Robledo *et al.*, 2026 ▸), could be employed to achieve a more accurate representation of the beam characteristics. However, for the purposes of this study, the divergence defined by the pinhole aperture and pinhole-to-sample distance provides a sufficiently realistic and practical basis for experimental design.

**Figure 3:**
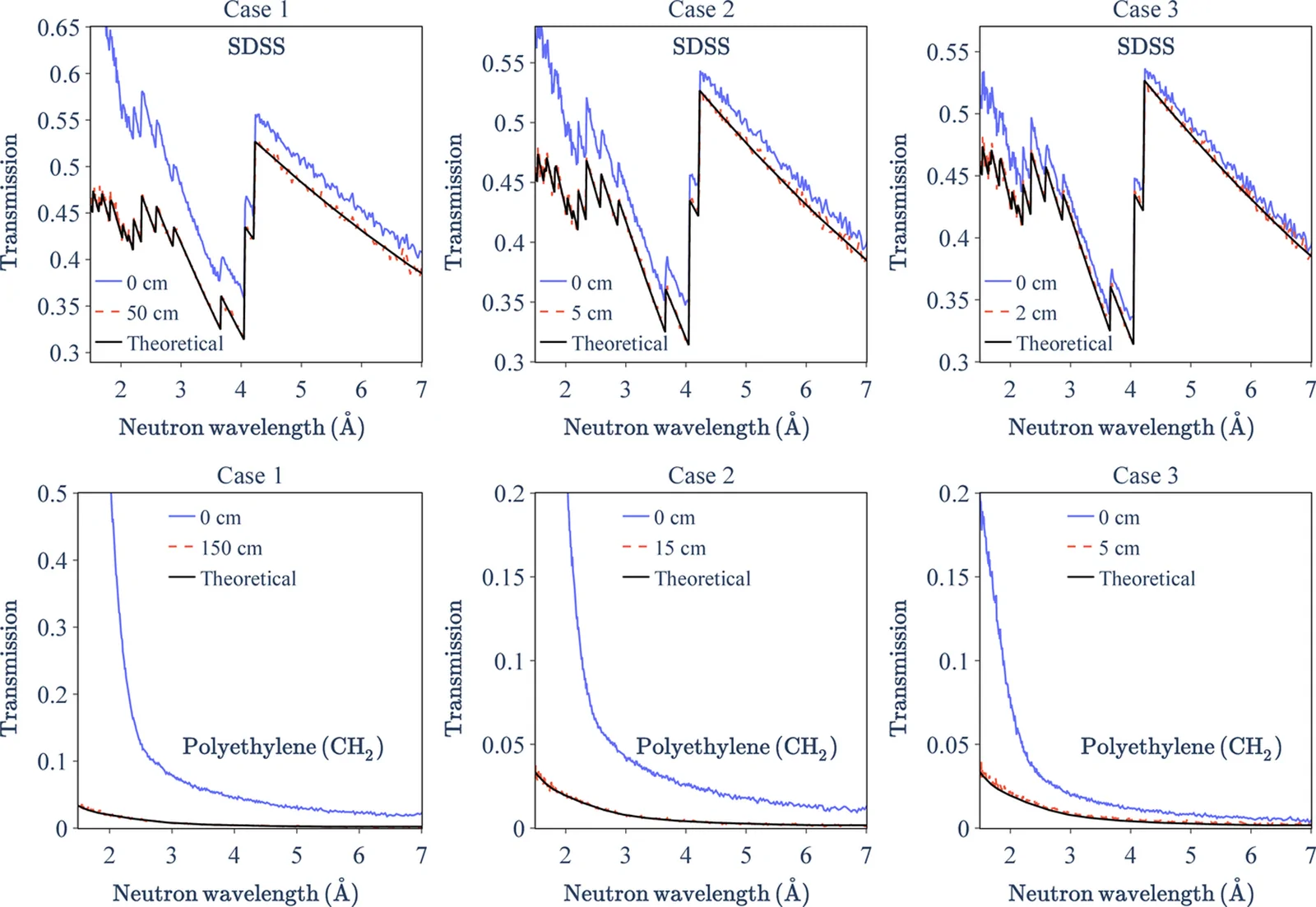
Comparison of simulated and theoretical transmission spectra (solid black lines) for 8 mm thick SDSS and PE across Cases 1, 2 and 3. Results indicate that sample-to-detector distances of 50 cm, 5 cm and 2 cm are required to mitigate forward-scattering artefacts for Cases 1, 2 and 3, respectively. In contrast, PE – a material with a high hydrogen content – requires significantly larger distances (150 cm, 15 cm and 5 cm) to eliminate these impacts. Additional spectra for other materials are provided in Figs. 13 and 14 in Appendix *A*[App appa].

The simulated beam profile with divergence is shown in Fig. 2 ▸(*a*). Regarding the theoretical transmission intensity *T*_th_(λ) for a sample composed of an ensemble of materials with each component indexed by *i*, *T*_th_(λ) can be calculated from each individual total neutron cross section σ_*i*_(λ), number density *N*_*i*_ and sample thickness *l*_*i*_, 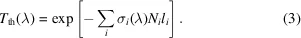
Fig. 3 ▸ compares simulated transmission spectra, at both a close distance (0 cm) and a sufficiently large sample-to-detector distance that yields data close to the theoretical neutron transmission predictions, for three different configurations, Cases 1, 2 and 3. The theoretical transmission spectra were derived from total cross sections calculated via *NCrystal* (Fig. 1 ▸) and equation (3), using nominal densities of 7.8 g cm^−3^ and 0.92 g cm^−3^ for SDSS and PE, respectively. Our results demonstrate that for an 8 mm thick SDSS sample, sample-to-detector distances of 50 cm, 5 cm and 2 cm are required for effective suppression of forward-scattering artefacts in Cases 1, 2 and 3, respectively. For comparison, we also considered 8 mm thick PE samples to represent materials with high hydrogen content. These simulations show that significantly larger sample-to-detector distances would be required in each case. A comparison of Cases 1 and 2 shows that a smaller slit size can dramatically reduce the required sample-to-detector distance (from 50 cm to 5 cm for the 8 mm thick SDSS) to mitigate forward-scattering effects.

Guided by these simulations, we selected an experimental configuration that minimizes forward-scattering effects while maintaining a sample geometry representative of conventional mechanical tensile testing. The investigated material consists of an SDSS specimen (gauge length 25 mm, thickness 8 mm, width 3 mm), partially covered with thin polyethylene (PE) sheets (length 2 mm, width 3 mm) of two different thicknesses (45 µm and 250 µm) to mimic hydrogen signals. The width of the specimen (3 mm) ensures that part of the neutron beam (with a 6.8 mm slit) remains unobstructed, providing an internal reference region for normalization of the transmission data. Theoretical transmission spectra for the SDSS specimen with and without PE attachments were calculated using equation (3).

**Figure 4:**
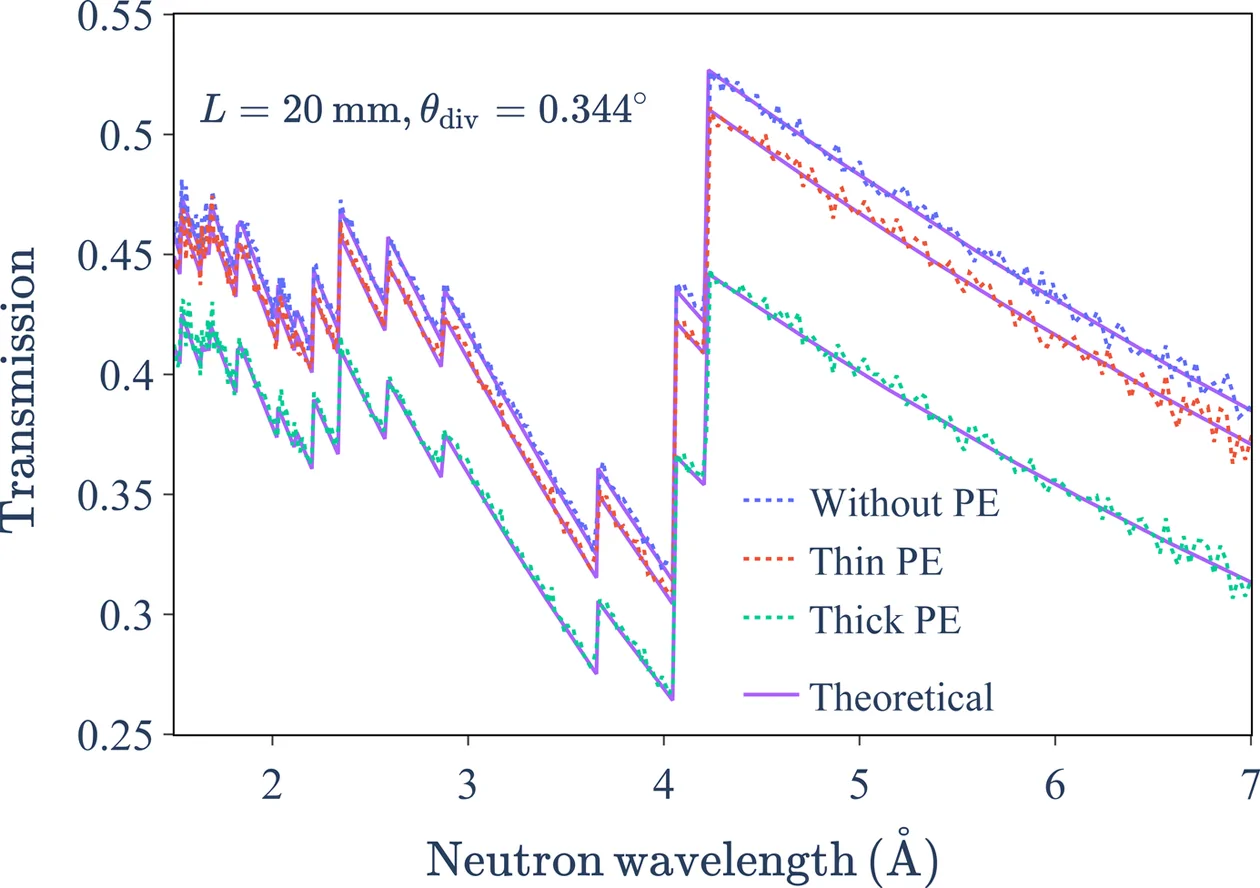
Comparison of simulated (*OpenMC*+*NCrystal*) *versus* theoretical transmission spectra for the front configuration (PE attachments facing away from the detector). For the Case 3 experimental setup, a sample-to-detector distance of 20 mm is sufficient to mitigate forward-scattering artefacts for an SDSS sample (25 mm in length of gauge, 8 mm thickness, 3 mm width), both with and without PE attachments, within the selected ROIs. Smaller sample-to-detector distances would introduce biases in the measured transmission spectra and thus hinder quantitative analysis. These data define the expected experimental results for the quantitative transmission measurements explained in this work.

We also performed *OpenMC*+*NCrystal* simulations to quantify potential forward-scattering effects for our specific experimental setup on IMAT (Case 3), which utilized a slit size of 21 × 6.8 mm. With a pinhole size of *D*_ph_ = 60 mm, the calculated divergence was θ_div_ = 0.344°. The regions of interest (ROIs) were configured to match the maximum regions usable for experimental data analysis: 8 × 3 mm for the SDSS grade without PE and 2 × 3 mm for the PE-attached regions. In practice, we restrict the ROIs to dimensions smaller than the sample to avoid neutron imaging edge enhancement effects due to different neutron refractive indices at the interfaces (Strobl *et al.*, 2009 ▸; Mirashi *et al.*, 2026 ▸); thus, the simulated transmission spectra represent the upper limits of potential forward-scattering artefacts. The results of the combined SDSS + PE simulations (Fig. 4 ▸) show that having the detector positioned 20 mm from the sample is still sufficient to mitigate forward-scattering artefacts.

Consequently, we adopted this configuration (Case 3 with a sample-to-detector distance of 20 mm) for our experimental setup on IMAT. Further discussion regarding the forward-scattering effects across the selected ROIs is detailed in Appendix *A*3.

## 4. Neutron imaging experimental setup and data processing

The methodology presented in this paper is established using experiments performed on the IMAT beamline at ISIS. The workflow was subsequently implemented on the RADEN beamline at J-PARC for the investigation of hydrogen-charged SDSS specimens. While the full RADEN study will be reported separately, reference measurements of the SDSS specimen with PE attachments are included here to give further validation of the sensitivity and reproducibility of the proposed methodology.

### 4.1. Wavelength-resolved imaging experiments on IMAT

**Figure 5:**
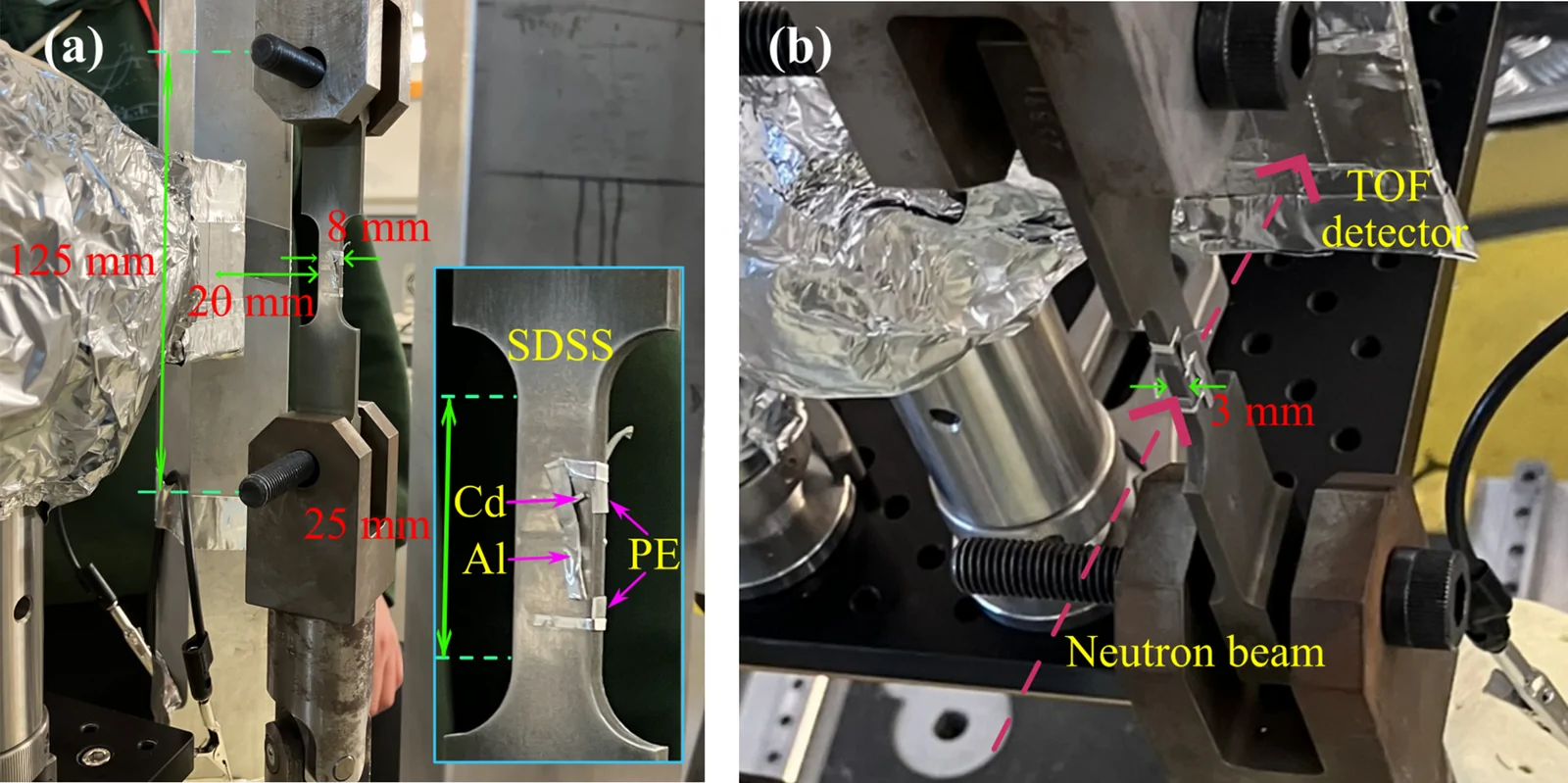
SDSS sample used in the transmission measurements conducted on the IMAT beamline at ISIS, (*a*) viewed perpendicular to the beam direction and (*b*) viewed along the beam direction. The dimensions of the SDSS sample (length of gauge, thickness, width = 25 × 8 × 3 mm) and the sample-to-detector distance (20 mm), as determined from Monte Carlo simulations, are indicated. Two PE sheets were attached to one surface of the SDSS sample using aluminium tape, to mimic known hydrogen concentrations, as shown in the enlarged image inset in panel (*a*). Neutron-absorbing cadmium markers were placed on the sides of the sample to aid subsequent image analysis. Not shown is that the entire sample was rotated by 180° for an additional measurement, placing the PE layers on the side facing the detector.

Neutron transmission measurements were performed on the IMAT beamline on an SDSS specimen. The sample was machined from bulk material (provided by the industrial collaborator Alleima) into a standard tensile geometry (Fig. 5 ▸), with a total length of 149 mm, a gauge length of 25 mm and a cross section of 8 × 3 mm. The nominal element compositions for the SDSS grade are given in Table 1 ▸. Two PE sheets with nominal thicknesses of 45 µm and 250 µm were attached to one surface using aluminium tape. The 250 µm sheet was sourced from Goodfellow (https://www.goodfellow.com) and had been previously characterized on the VESUVIO spectrometer at ISIS. Cadmium markers were used to indicate the positions of both PE sheets. As hydrogen is commonly quantified in parts per million (wt p.p.m.), the two PE layers (density 0.92 g cm^−3^) correspond to hydrogen-equivalent concentrations of approximately 95 wt p.p.m. (45 µm sheet) and 530 wt p.p.m. (250 µm sheet) relative to the underlying steel volume.

The experimental setup employed a LumaCam detector system (Losko *et al.*, 2021 ▸; Wolfertz *et al.*, 2024*a* ▸) positioned downstream of the neutron beam path. Using optical lenses, the system allows for an adaptable field of view (active detector area). As the experiment targeted small changes in the transmission signal, it was essential to use a detector with low gamma sensitivity and high dynamic range to minimize uncertainties in the measurements. The LumaCam fulfils these requirements through on-line single neutron event reconstruction in counting mode, enabling the discrimination of individual neutron interactions within the scintillator. This approach suppresses scintillator afterglow and background contributions, resulting in a detector system with high spatiotemporal resolution suitable for a wide range of neutron imaging applications (Wolfertz *et al.*, 2024*b* ▸; Jäger *et al.*, 2024 ▸; Jäger *et al.*, 2025 ▸; Mirashi *et al.*, 2025 ▸; Hirsh *et al.*, 2025*b* ▸; Khaplanov *et al.*, 2025 ▸; Hirsh *et al.*, 2025*a* ▸). Individual neutron events can be identified through the nanosecond resolution of their time of arrival. This facilitates the separation of two neutron events that have different arrival times. A limitation of this method is that the neutron intensity must not exceed a threshold where individual neutron events can no longer be temporally resolved. An alternative detector technology with high spatiotemporal resolution is the neutron-sensitive microchannel plate (MCP) detector (Tremsin & Vallerga, 2020 ▸).

Using the LumaCam detector in this experiment, a field of view of 28 × 28 mm was configured and binned into 512 × 512 pixels in space (55 µm effective pixel size). The time-of-flight (TOF) binning was set to 25 µs bin width, resulting in 4000 individual images across the wavelength spectrum for each individual measurement. The pinhole size of IMAT was set to 60 mm, corresponding to an *L*/*D* ratio of 935. Initial calibration measurements were performed with 15 min acquisition time for both the open-beam and an iron powder reference sample, corresponding to proton charges of 10.1023 µAh and 10.0602 µAh, respectively. The TOF–wavelength relationship is given by 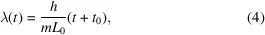
where *t* is the TOF, *h* is Planck’s constant, *m* is the neutron mass, *L*_0_ is the flight path length and *t*_0_ is the time delay of the source trigger.

The determined flight path length is *L*_0_ = 56.1 (3) m and the time delay is 3.2 µs. Using the TOF–wavelength relationship, the measured open-beam spectrum can be determined.

The subsequent wavelength-resolved transmission data fitting excluded image frames of neutron wavelength λ ≤ 1.6 Å, due to a poor signal-to-noise ratio, and those of λ ≥ 7 Å, where the neutron flux diminishes significantly.

**Figure 6:**
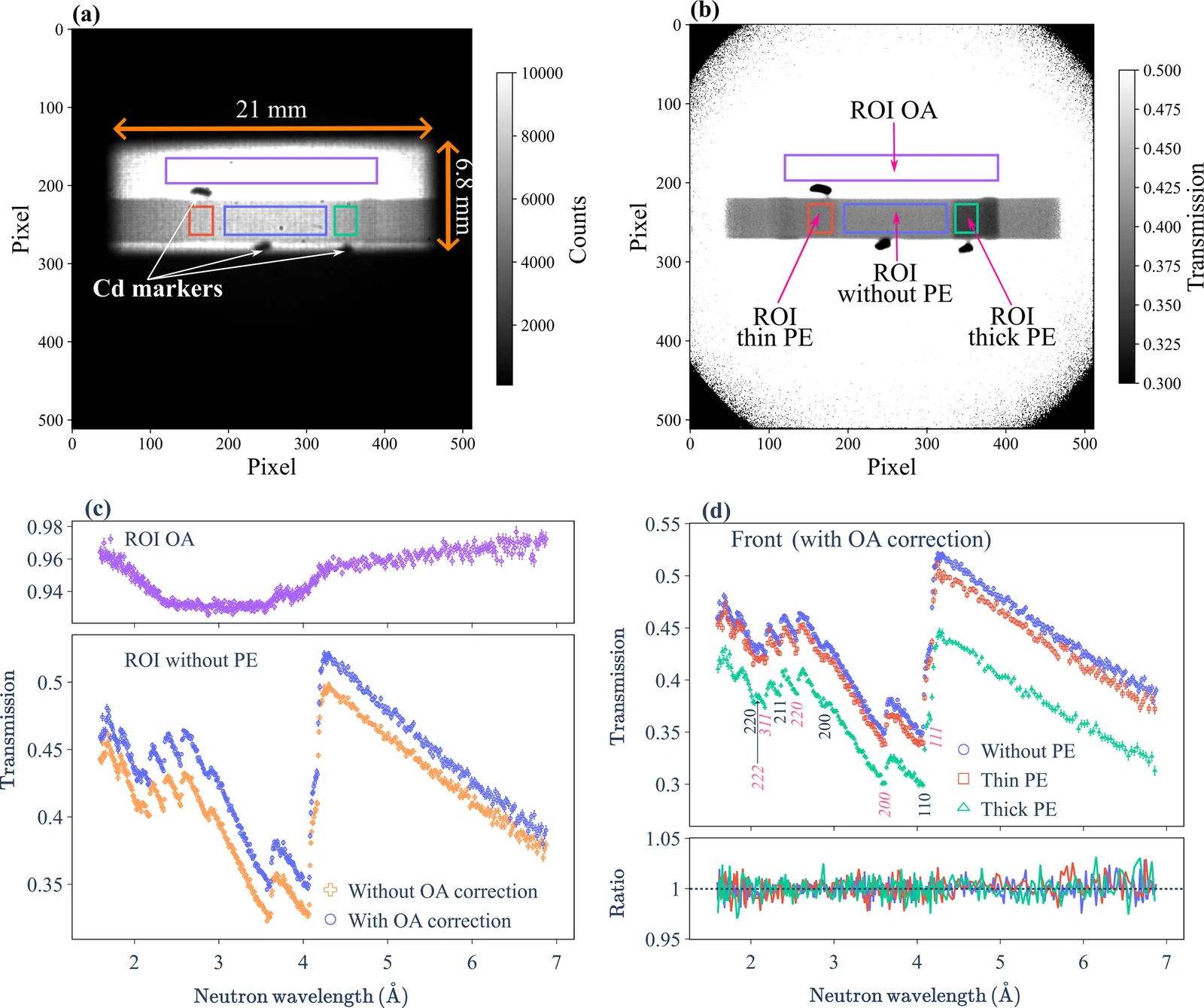
(*a*) Integrated neutron radiograph of the front SDSS sample configuration, summed over the wavelength range of 1.6–7.0 Å. The 21 × 6.8 mm rectangular beam profile, the sample and three cadmium markers are clearly visible within the 512 × 512 pixel detection area. (*b*) Normalized transmission image obtained by dividing the image in panel (*a*) by the open-beam reference (integrated over the same wavelength range) with proton-charge normalization. Four ROIs [coloured red, blue, green and purple, also indicated in (*a*)] were defined based on cadmium marker positions: thin PE attachment (left), without PE attachments (bottom centre), with thick PE attachment (right) and OA (top centre). (*c*) Front neutron transmission spectra for the OA (top panel) and the PE-free SDSS ROI (lower panel). The OA correction compensates for the wavelength-dependent offset observed in the OA transmission, resulting in a non-negligible upward shift of the transmission spectrum. (*d*) Transmission spectra for all ROIs after OA correction. Primary *hkl* reflections for both the ferrite and austenite phases are labelled (austenite indices in italics). Back spectra are omitted due to their overlap with front measurements. The lower panel displays the front-to-back transmission ratios, showing a consistent average ratio of unity across all ROIs.

Guided by the preceding *OpenMC*+*NCrystal* simulations, we adopted the Case 3 configuration for the experiment, positioning the SDSS specimen (attached to two PE sheets of differing thicknesses) at a distance of 20 mm from the detector. The distance is defined from the end of the sample to the detector sensor. Two complementary measurements were performed: (i) a ‘front’ configuration with the PE sheets facing the beam (Fig. 5 ▸) and (ii) a ‘back’ configuration with the sample flipped 180°, placing the PE sheets downstream. Acquisition times were 46 min for the front configuration and 61.5 min for the back configuration, corresponding to integrated proton charges of 30.258 and 40.119 µAh, respectively. Fig. 6 ▸(*a*) displays the front radiograph integrated across neutron wavelengths of 1.6–7.0 Å, confirming proper sample alignment within the 21 × 6.8 mm beam profile. Four rectangular ROIs, delineated in red (thin PE), blue (without PE), green (thick PE) and purple [open area (OA)], were defined based on cadmium marker positions. The OA serves as a reference to monitor neutron intensity fluctuations or other systematic variations.

Following the same integration procedure for open-beam measurements (155 min, 101.603 µAh), we obtained a normalized transmission image by dividing the sample radiograph by the open-beam data, with both datasets normalized by proton charge. Fig. 6 ▸(*b*) presents the resulting transmission image; the distinct transmission intensities across the three ROIs validate the selection of these regions. To derive wavelength-dependent spectra, detector counts within each ROI were summed per time frame and normalized by the corresponding open-beam counts and proton charges. The upper panel of Fig. 6 ▸(*c*) displays the resulting spectrum in the OA region for the front configuration. The observed transmission is up to 7% below unity, although it should theoretically be 1, indicating an additional source of intensity fluctuation. Because the measured transmission is below unity, scattering from the sample into the OA can be excluded, as such scattering would instead increase the detected intensity. A more plausible explanation is detector-related parasitic scattering that contributes to the open-beam measurement. During the sample measurement, part of the direct neutron beam that contributes to this detector-related background is attenuated by the sample. Consequently, the detector-generated background is reduced in the sample measurement compared with the open-beam measurement, leading to an apparent transmission below unity after normalization.

Although this interpretation cannot be proven directly from the present measurements, it is supported by the comparison with the RADEN measurements presented below, where a different detector configuration exhibits OA transmission much closer to unity. To compensate for this wavelength-dependent offset, we implemented an OA-based correction by dividing each transmission spectrum by the wavelength-dependent OA intensity.

The corrected transmission spectra are shown in [Fig. 6 ▸(*d*)]. They allow for the clear identification of Bragg edges corresponding to the ferrite (b.c.c.) and austenite (f.c.c.) phases. As expected, the PE-attached regions show reduced transmission compared with PE-free areas. This attenuation scales with PE thickness and a detailed quantitative analysis is presented later in this work. Despite the smaller ROIs for the PE-covered regions, the propagated statistical uncertainties remain sufficiently low. The back transmission spectra overlap with the front spectra. The ratios between them are near to unity, as shown in the lower panel.

### 4.2. Wavelength-resolved imaging experiments at RADEN

**Figure 7:**
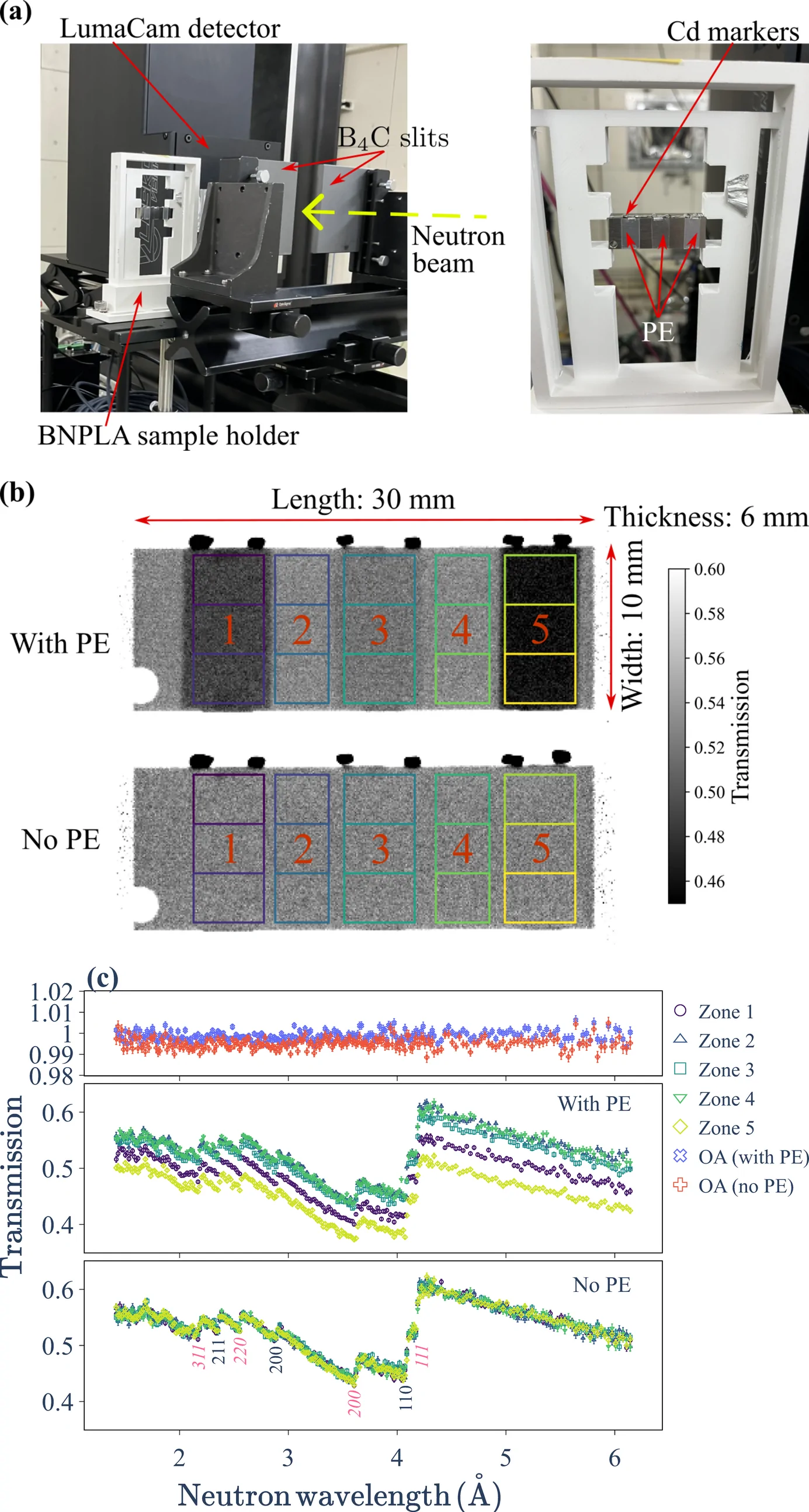
(*a*) Experimental setup for wavelength-resolved neutron imaging on the RADEN beamline at J-PARC. The sample holder is moved to be irradiated by the neutron beam during measurements. Three PE sheets with different nominal thicknesses of 100, 35 and 230 m (left to right) are attached to the 6 mm thick SDSS sample using aluminium tape. Cadmium markers indicate the PE sheet positions. (*b*) Neutron transmission images of the SDSS sample with and without the PE sheets. Fifteen selected ROIs are arranged in a 5 column × 3 row grid. Five zones in the second row are labelled with numbers from 1 to 5. (*c*) Transmission spectra for the OA regions (top) and for the second-row ROIs (Zones 1 to 5) of the SDSS sample with and without PE sheets (middle and bottom). Primary *hkl* reflections for both the ferrite and austenite phases are labelled (austenite indices in italics).

To demonstrate the transferability of the proposed workflow, the methodology developed for the IMAT measurements was directly applied to experiments on the RADEN beamline at J-PARC (Shinohara *et al.*, 2020 ▸). Specimens with dimensions of 30 × 10 × 6 mm from the same SDSS material, covered with three PE sheets of nominal thicknesses 35, 100 and 230 µm, were measured. As in the IMAT study, *OpenMC*+*NCrystal* simulations were first performed to optimize the experimental geometry. A sample-to-detector distance of 50 mm was found to be sufficient to eliminate forward-scattering effects across the complete 30 × 10 mm ROI. The experimental setup is shown in Fig. 7 ▸(*a*). Great care was taken to define the neutron beam upstream of the sample using several neutron-absorbing beam limiters (made from B_4_C). The sample holder was 3D-printed from neutron-absorbing BNPLA [mixing of hexagonal boron nitride (h-BN) and polylactic acid (PLA)] (Sebold *et al.*, 2024 ▸) and designed to minimize the amount of material surrounding the sample while maximizing the air volume around it, thereby reducing parasitic neutron scattering. The PE sheets (with a density of 0.92 g cm^−3^) were sourced from Goodfellow (https://www.goodfellow.com) and were in pristine condition, without any previous exposure to neutron radiation.

The LumaCam detector was configured with a field of view of 47 × 47 mm and operated in event-counting mode, recording 512 × 512 pixels and 2000 individual TOF images per pulse with a time-bin width of 20 µs.

The TOF–wavelength relationship was calibrated using an iron powder measurement, yielding a flight path length of 23.7 (1) m and a time delay of 3.8 µs via equation (4). Three actual measurements were then conducted: open beam, the SDSS sample with PE sheets and the SDSS sample without PE sheets. The exposure time of each measurement was 20 min, corresponding to trigger counts (used at RADEN instead of accumulated proton charge) of 25828, 25827 and 25827, respectively. Fig. 7 ▸(*b*) shows the resulting neutron transmission images, obtained by dividing the integrated radiograph of the sample by that of the open beam and normalizing by their respective trigger counts. Fifteen ROIs were selected and arranged in a 5 × 3 (column × row) grid based on the PE sheet positions. The corresponding transmission spectra of the second row of the ROIs, labelled with numbers from 1 to 5, corrected for OA (transmission spectra shown in the top panel), are shown in Fig. 7 ▸(*c*). The OA transmission spectra are below but much closer to unity than those measured on IMAT. This observation further supports the interpretation that the wavelength-dependent offset originates from a detector-related systematic effect rather than changes in the incident neutron spectrum. While the exact origin of the detector-related background cannot be identified conclusively from the present measurements, the different detector configuration at RADEN evidently reduces this effect, resulting in OA transmission values much closer to unity. These results highlight the importance of carefully controlling detector-related systematic effects for highly quantitative wavelength-resolved neutron transmission measurements.

## 5. *NCrystal*-based least-squares fitting

### 5.1. Data analysis procedure

**Figure 8:**
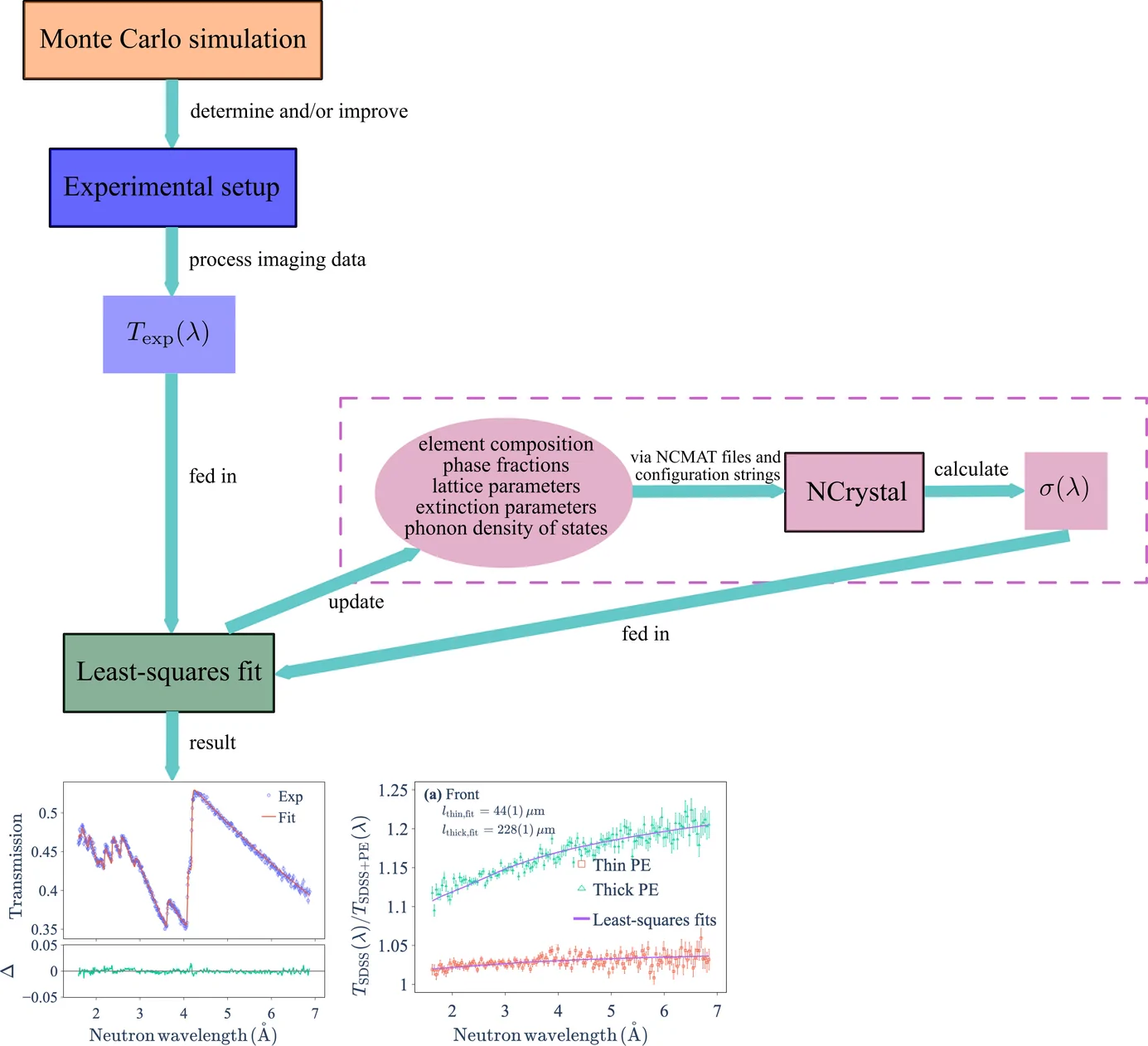
Overall workflow developed in this work, which includes Monte Carlo simulations to determine the experimental configuration, data processing and a least-squares fitting procedure based on the cross section σ(λ) calculated by *NCrystal*. See text for a detailed explanation.

This section details our least-squares fitting procedure, built upon the *NCrystal* toolkit, for the quantitative analysis of wavelength-resolved neutron imaging data. The procedure is implemented in a script that has been made publicly available (Xu, 2025 ▸). The overall workflow of the present study is summarized in Fig. 8 ▸. The determination of the experimental setup using Monte Carlo simulations and the subsequent data processing have been detailed in Sections 3 and 4, respectively. *NCrystal* calculates the wavelength-dependent total neutron cross section [equation (1)] by incorporating key microstructural parameters: elemental composition, phase fractions, unit-cell parameters, extinction-related parameters and PDOS. These properties are specified via NCMAT files, built-in configuration strings or the specialized *CrysExtn* plugin (Xu, 2024 ▸; Xu *et al.*, 2025 ▸) for extinction effects. Notably, the extinction model for spherical crystallites has recently been improved numerically (Kittelmann *et al.*, 2026 ▸) and will be integrated as a built-in functionality within *NCrystal*.

The wavelength-dependent neutron cross section calculated by *NCrystal* provides a forward model for analysing the transmission spectrum. The analytical transmission intensity, *T*(λ), is calculated by convoluting the theoretical transmission intensity *T*_th_(λ), given in equation (3), with the resolution function *R*(λ), 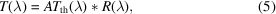
where *A* is a scale factor (typically near unity) to account for systematic variations such as residual wavelength-independent normalization differences. Even though *A* is considered flat across the wavelength range used in this study, it can be further refined using a more complex model.

For the IMAT beamline, we found that a Jorgensen-type resolution function (Jorgensen *et al.*, 1978 ▸; Von Dreele *et al.*, 1982 ▸) provides an excellent fit to the Bragg edge transmission spectrum. This function is characterized by seven free parameters: α_0_, α_1_, β_0_, β_1_, Σ_0_, Σ_1_ and Σ_2_. The parameters α and β (parameterized by α_0_ and α_1_, β_0_ and β_1_, respectively) are the exponential rise and decay constants that characterize the asymmetric line shape of the neutron moderator pulse. The parameter Σ (parameterized by Σ_0_, Σ_1_ and Σ_2_) represents the standard deviation of the Gaussian component of the line shape, which originates from instrumental (Σ_0_ and Σ_1_) and sample strain (Σ_2_) contributions (Sabine *et al.*, 1988 ▸). Following established conventions (Vogel, 2000 ▸), and considering that our samples are strain-free, α_0_, Σ_0_ and Σ_2_ were set to zero in our analysis. We detail the determination of the Jorgensen function parameters for the IMAT beamline in Appendix *A*5. While this function can also model the resolution of the RADEN beamline, determining its parameters is beyond the scope of this work, as the primary objective of the RADEN data is fitting the PE thickness from spectral ratios. This parameter determination for RADEN will be addressed in a future study.

A primary objective of this study is the quantitative detection of trace materials, specifically low-concentration hydrogen within metals, which is simulated using PE layers in the present work. To isolate the attenuation contribution of the PE sheets, the transmission spectrum of the base material *T*_SDSS_(λ) is divided by the spectrum of SDSS and PE, *T*_SDSS+PE_(λ). According to the Beer–Lambert law, the ratio is obtained by 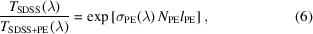
where σ_PE_(λ) is the total neutron cross section of PE [Fig. 1 ▸(*b*)], *N*_PE_ = 0.1185 atoms Å^−3^ is the number density (calculated from a mass density of 0.92 g cm^−3^) and *l*_PE_ is the sample thickness, which is determined by fitting the spectral ratio.

In practice, critical material parameters, such as precise phase fractions and unit-cell parameters, are often not fully known *a priori*. To address these challenges when analysing full transmission spectra, we developed a nonlinear least-squares fitting routine using the Python package *lmfit* (Newville *et al.*, 2024 ▸). This routine minimizes the residual between the experimental transmission spectrum *T*_exp_(λ) and the analytical spectrum *T*(λ), or the experimental and analytical ratios [equation (6)], as calculated by *NCrystal*. Notably, while fitting the PE thickness is not strictly nonlinear, the overall transmission spectrum fitting remains inherently nonlinear because the underlying parameters (*e.g.* phase fraction and scale factor) do not share a linear correlation. The subsequent sections detail the fitting procedure and present the results for the SDSS spectra and the PE thickness.

### 5.2. Results and discussion

#### 5.2.1. IMAT measurements

The transmission spectrum of the SDSS sample without PE attachments, measured in the front configuration, was analysed first. To ensure robust convergence of the fitting procedure, we fixed the values of well defined parameters associated with the transmission calculation. These fixed parameters included the sample thickness (8 mm) and density (7.8 g cm^−3^), the temperature (296 K) and the element composition specified in Table 1 ▸. Additionally, the flight path length (56.1 m) and time delay (3.2 µs) were fixed using values determined from the iron powder calibration measurements described above in Section 4.

Unit-cell parameters (2.88 Å for the ferrite phase and 3.61 Å for the austenite phase) were fixed based on the Bragg edge positions in the transmission spectrum. Although dissolved hydrogen can induce lattice expansion in steels, an effect that has been investigated for SDSS in recent modelling and diffraction studies (Lindblom *et al.*, 2026 ▸), the present study focuses on hydrogen-equivalent signals introduced via external PE layers, and the unit-cell parameters were kept fixed during refinement. For the extinction model, following the assumptions of Xu *et al.* (2025 ▸), the grain size parameter in the model was set equal to the average value measured by electron backscatter diffraction (EBSD). In this work, the average grain sizes measured by EBSD for the ferrite and austenite phases were 17 µm and 12 µm, respectively; these values were kept fixed in the extinction model during fitting.

The Jorgensen-type resolution function parameters were determined as listed in Fig. 17 in Appendix *A*5 and kept constant. The remaining free parameters for the SDSS grade (front configuration, no PE attachments) and their fitted values are listed in Table 2 ▸.

**Figure 9:**
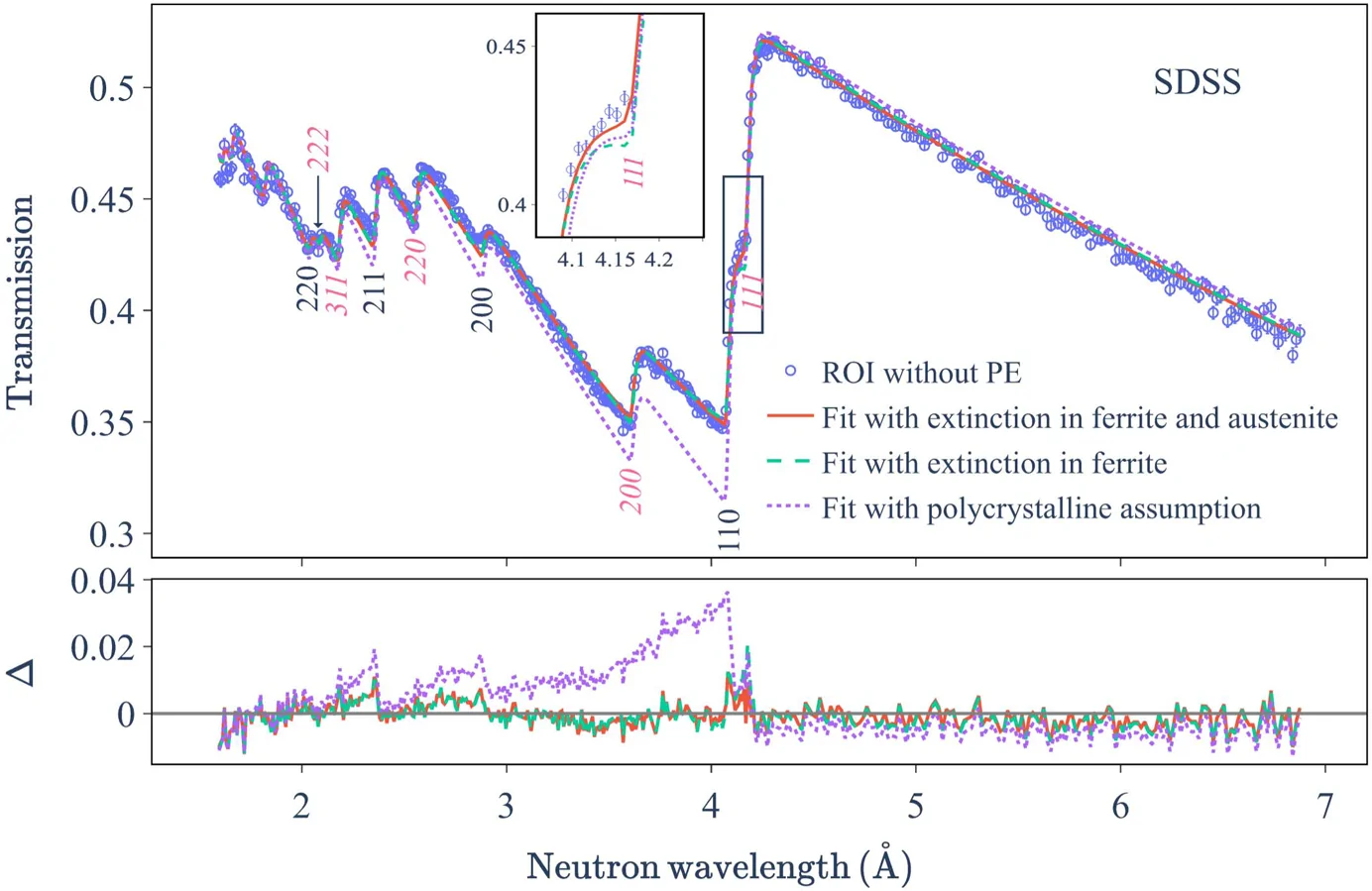
*NCrystal*-based least-squares fits of the SDSS grade within the ROI without PE attachments [blue zone in Fig. 6 ▸(*a*)]. Three fitting scenarios are presented: the dotted line assumes an ideal polycrystalline state, the solid line accounts for extinction effects in both the ferrite and austenite phases, and the dashed line accounts for extinction effects exclusively in the ferrite phase. The 111 reflection is highlighted, indicating that the fit is improved when secondary extinction effects are considered in the austenite phase. Main *hkl* reflections for both ferrite and austenite phases are labelled, with austenite phase indices italicized. See text for further discussion.

As shown in Fig. 9 ▸, the quality of the fit improves significantly when the Becker & Coppens extinction model (Becker & Coppens, 1974*a* ▸; Becker & Coppens, 1974*b* ▸) is applied to both phases, compared with the assumption of an ideal polycrystalline material. Primary and secondary extinction effects were considered for the ferrite phase, while only type-I secondary extinction was applied to the austenite phase. The reduced χ^2^ value drops from 50 (no extinction) to 3.8 (with extinction in ferrite and austenite). Restricting the extinction model exclusively to the ferrite phase yielded a comparable fit quality (with a reduced χ^2^ value of 4.3), suggesting that extinction effects are predominant in the ferrite phase of our SDSS sample. The observed extinction in the ferrite phase aligns with observations by Santisteban *et al.* (2001 ▸), who attributed transmission data discrepancies in b.c.c. iron powder to extinction effects. Notably, mosaic spread parameters cause a rounding of the Bragg edges, which is correlated with the Jorgensen-type resolution function of the instrument. Therefore, prior determination of the resolution parameters (Fig. 17) decoupled these effects, enabling a more precise quantification of the extinction parameters.

A full quantification of these extinction effects would require complementary diffraction measurements to distinguish them from potential texture, as demonstrated by Xu *et al.* (2025 ▸). While this suggests an interesting path for future research, the primary objective of this work is to establish a general analysis pipeline for wavelength-resolved neutron imaging. Therefore, a detailed analysis of extinction in iron remains beyond the immediate scope of this paper.

So far, the full transmission spectra have been analysed, including contributions from unit-cell parameters, element composition and extinction effects. However, if the primary objective is solely to quantify hydrogen, a simplified and more robust approach can be employed by considering the ratio of transmission spectra, as introduced in equation (6). In this approach, the signal from a reference region without hydrogen (or without PE) is divided by that of a hydrogen-containing region, effectively isolating the hydrogen contribution while largely cancelling out systematic effects originating from the sample and instrument.

**Figure 10:**
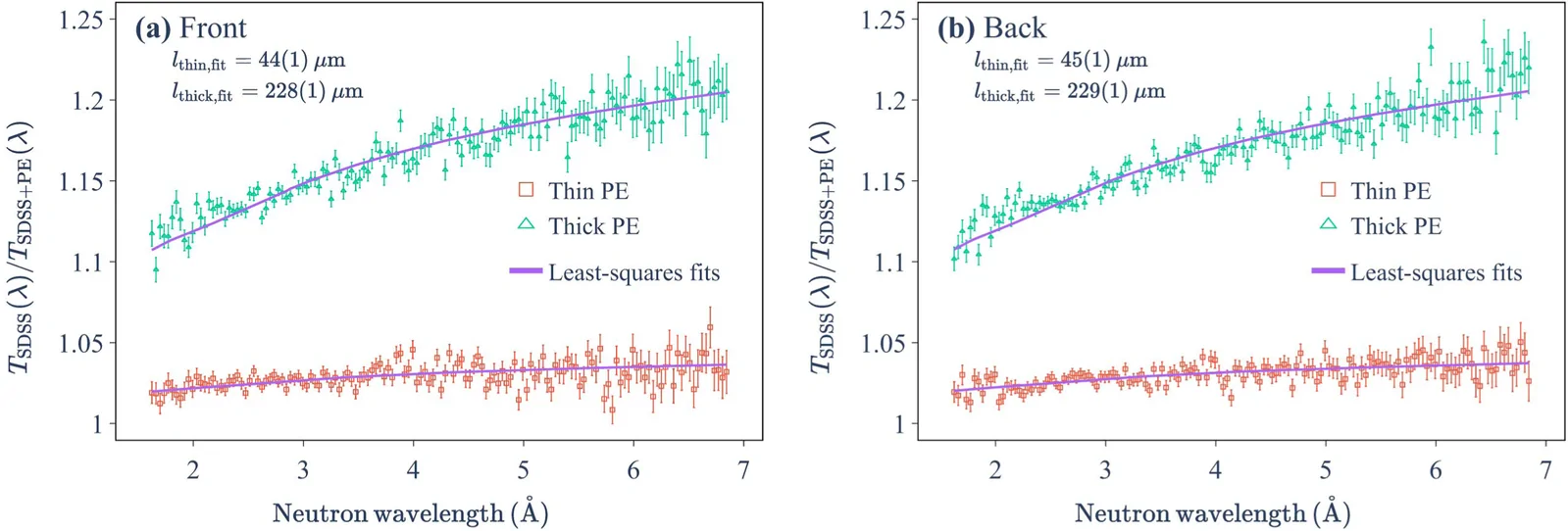
Experimental (markers) and fitted (lines) neutron transmission spectra ratios for the SDSS sample measured on IMAT: (*a*) front and (*b*) back measurements. The *NCrystal*-based least-squares fits quantitatively reproduce this attenuation trend, yielding PE thicknesses with 1 µm uncertainty, as listed. See text for further discussion.

For this analysis, the density of PE was fixed at 0.92 g cm^−3^ and we used the σ_PE_(λ) data in Fig. 1 ▸(*b*) as numerical values for each wavelength bin. The resulting transmission ratios are shown in Fig. 10 ▸ for both front and back configurations. The corresponding fits show excellent agreement with the experimental data. The fitted PE thicknesses are consistent between both configurations, yielding *l*_thin,fit_ = 44 (1) µm and *l*_thick,fit_ = 228 (1) µm for the front, and *l*_thin,fit_ = 45 (1) µm and *l*_thick,fit_ = 229 (1) µm for the back configuration.

Given the nominal PE sheet thicknesses of 45 µm and 250 µm, our results show excellent agreement for the thin PE but underestimate the thick sheet by approximately 10%. Discussions regarding the discrepancy of the thick PE thickness, which may stem from the reduced hydrogen-to-carbon (H/C) ratio in this particular sample, are detailed in Appendix *A*6.

#### 5.2.2. RADEN measurements

Section 5.2.1 details the data analysis procedure for the IMAT experiment, which includes both nonlinear least-squares fitting on wavelength-resolved transmission spectra and spectral ratio fitting to determine PE thickness. In contrast, the subsequent RADEN data analysis focuses specifically on fitting the PE thickness.

**Figure 11:**
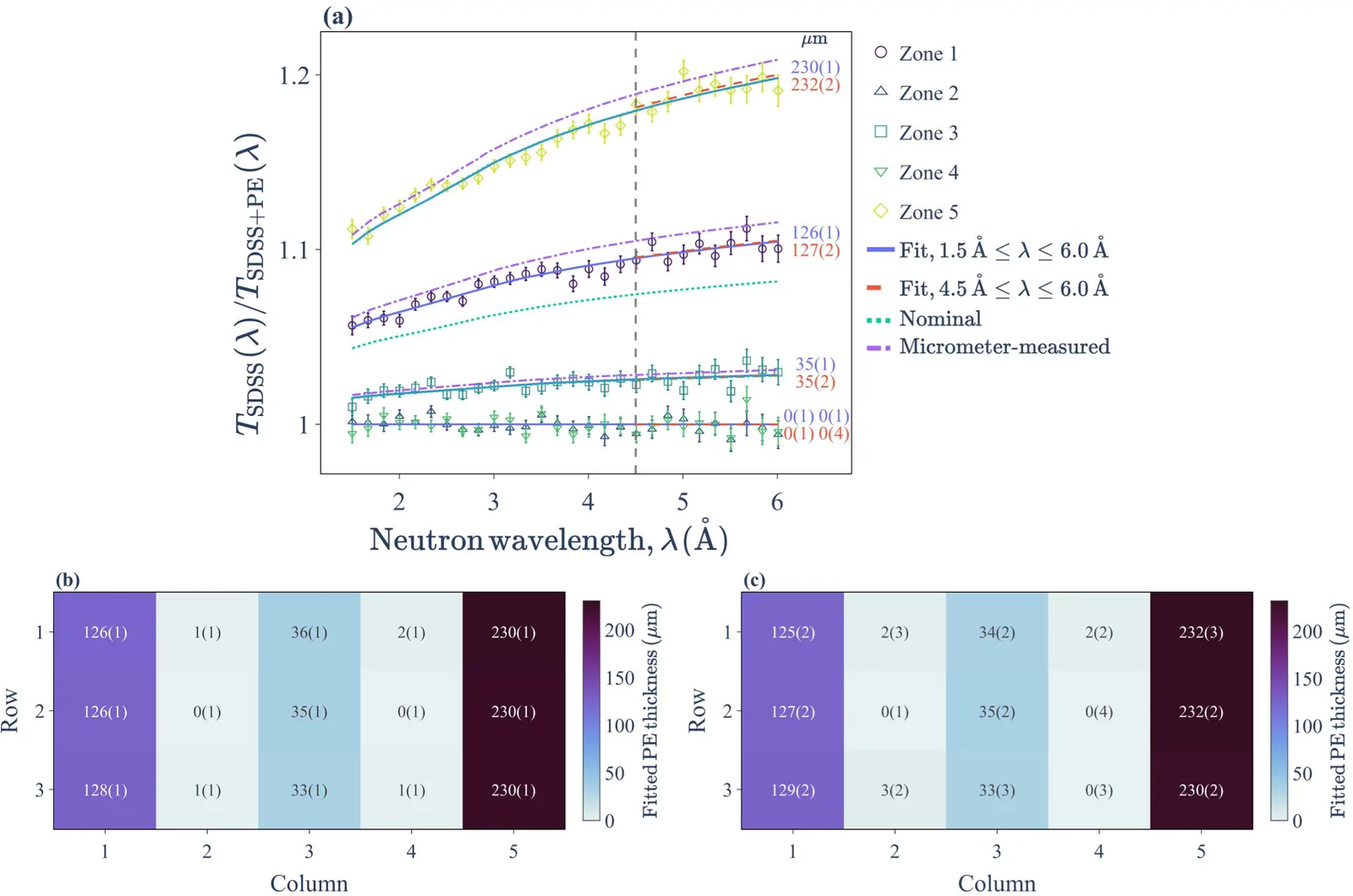
(*a*) Experimental and fitted neutron transmission spectra ratios for Zones 1–5. Experimental data (binned for clarity) are shown alongside fits over the full wavelength range (1.5–6.0 Å, solid lines) and after the Bragg cutoff (4.5–6.0 Å, dashed lines). Inset values indicate the corresponding fitted PE thicknesses. For comparison, the transmission ratios calculated using nominal (dotted lines) and micrometer-measured (dashed–dotted lines) PE thicknesses are also shown. (*b*) and (*c*) Two-dimensional fitted PE thickness maps for the wavelength ranges of 1.5–6.0 Å and 4.5–6.0 Å, respectively. See text for further discussion.

**Figure 12:**
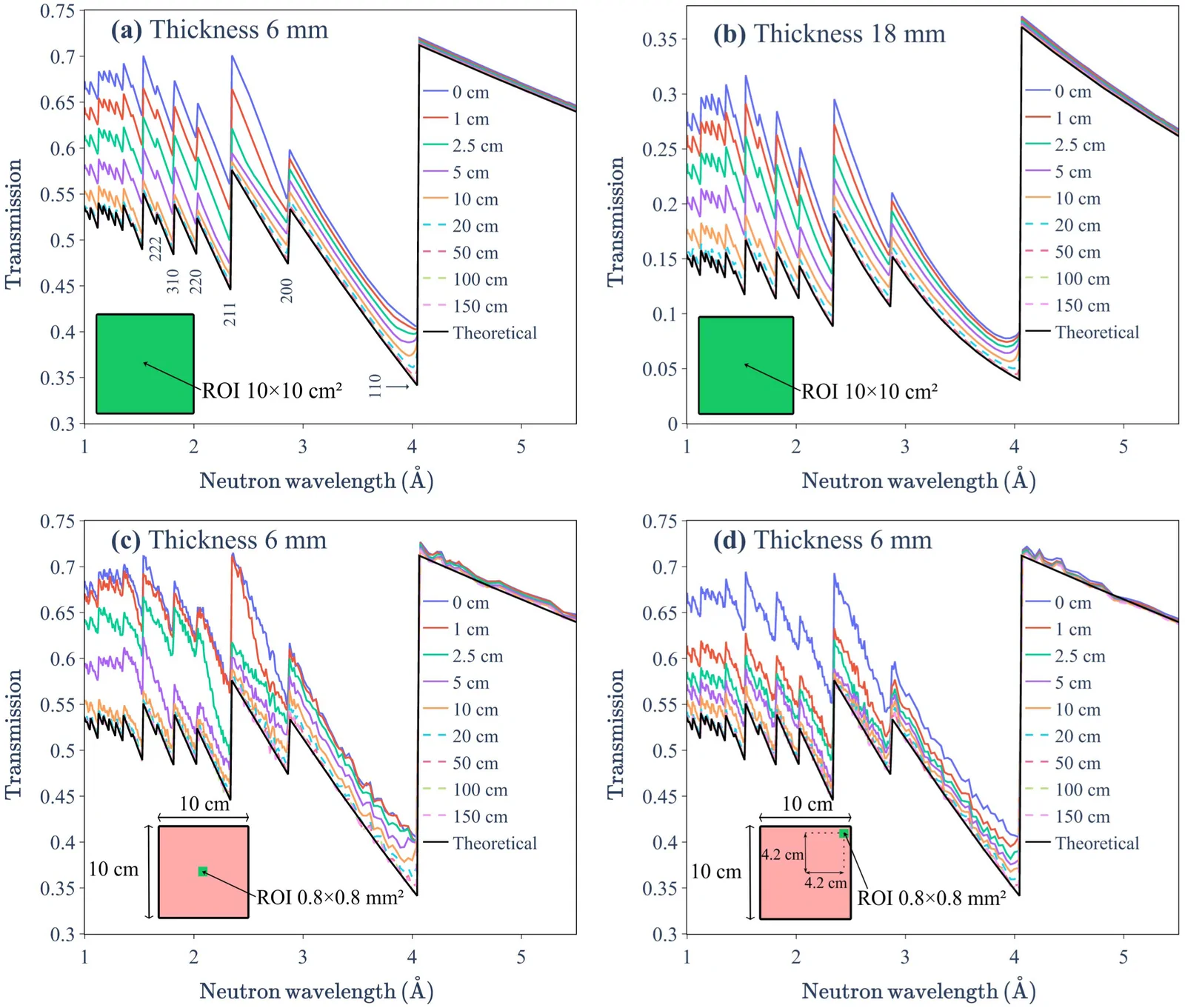
*OpenMC*+*NCrystal* simulated transmission spectra for a b.c.c. iron plate illuminated by a 10 × 10 cm parallel neutron beam. The setup replicates the configurations presented by Sato *et al.* (2017 ▸). Spectra are shown for a TOF detector positioned at various distances downstream from the sample; theoretical predictions are indicated by solid black lines. Iron plate thicknesses and the sizes and positions of the ROIs are specified in each panel. The results in panels (*a*), (*c*) and (*d*) are in good agreement with Figs. 2, 5 and 6, respectively, in the work of Sato *et al.* (2017 ▸), validating our *OpenMC*+*NCrystal* simulation framework.

**Figure 17:**
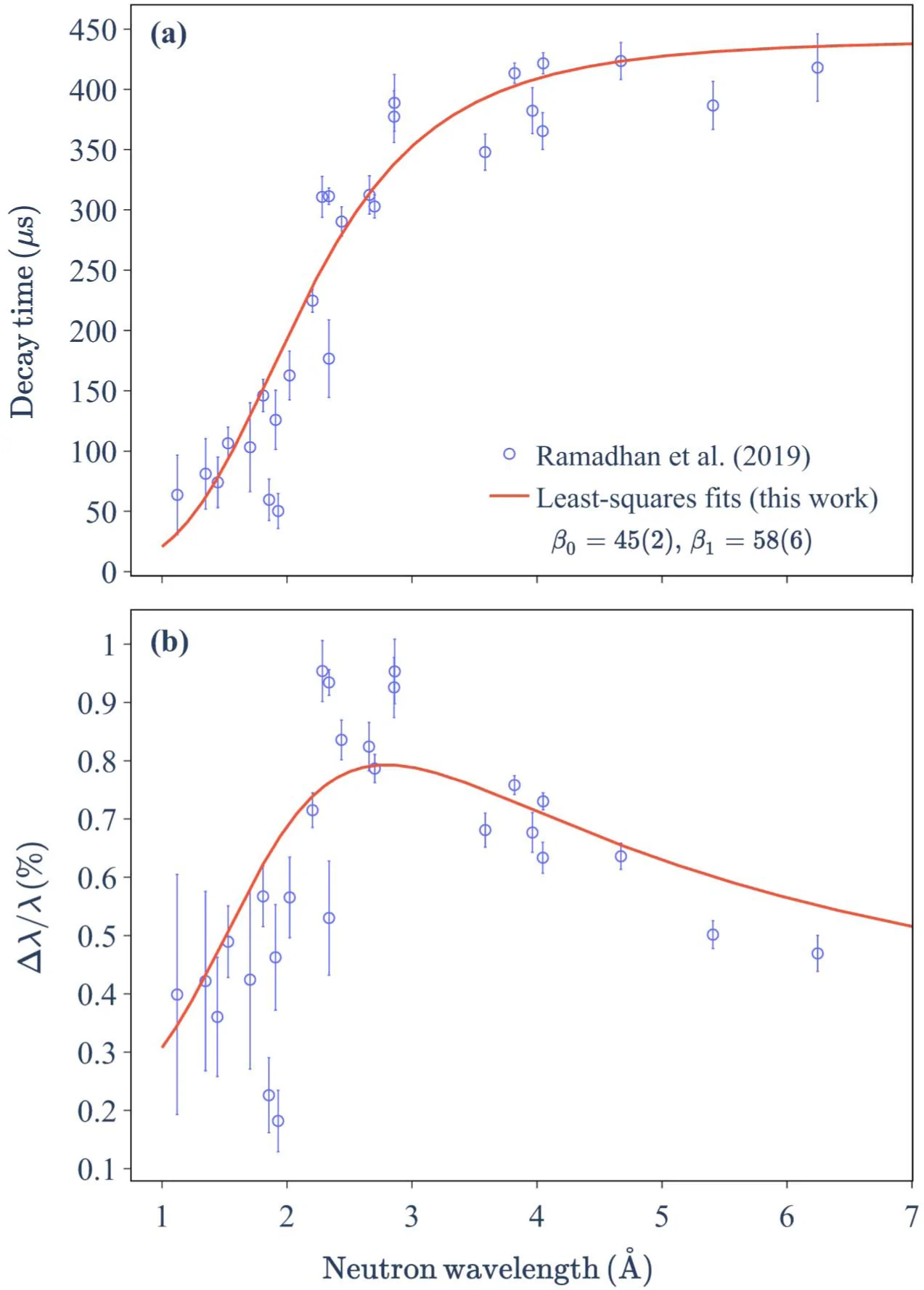
(*a*) Decay time τ and (*b*) resolution as a function of neutron wavelength on IMAT. The experimental data shown in both panels were digitized from Ramadhan *et al.* (2019 ▸) and they are fitted simultaneously using equations (8) and (9). The parameters β_0_ and β_1_ determined from nonlinear least-squares fitting are listed. See text for further discussion.

Transmission ratios were obtained by dividing the spectra of the SDSS sample without PE by those with PE for Zones 1 to 5 shown in Fig. 7 ▸(*c*). The resulting ratios for the selected ROIs are shown in Fig. 11 ▸(*a*). Following the fitting procedure detailed above, the PE thicknesses were fitted across two distinct intervals: the full neutron wavelength range (1.5–6.0 Å) and the range after the Bragg cutoff (4.5–6.0 Å). The fitted thicknesses and their associated uncertainties are listed in the figure. Notably, the fitted PE thicknesses obtained from both wavelength ranges are consistent within their uncertainties.

The reason for performing a fit after the Bragg cutoff stems from the fact that hydrogen can induce lattice expansion in steels, as recently studied by *e.g.* Lindblom *et al.* (2026 ▸). Additionally, hydrogen could potentially change the extinction and/or texture effects. These influences might distort the transmission ratio prior to the cutoff. Nevertheless, incorporating the lower-wavelength data remains critical for investigating the dynamics of hydrogen in dual-phase steels.

By applying this analysis systematically, we generated 2D thickness maps fitted for both the 1.5–6.0 Å and 4.5–6.0 Å ranges, as shown in Figs. 11 ▸(*b*) and 11 ▸(*c*), respectively. The fitted PE thicknesses in the middle and rightmost regions are in excellent agreement with their nominal values. However, the fitted PE thickness on the left is approximately 30% higher than its nominal value. To investigate this, the PE sheets were measured using a Mitutoyo Digimatic micrometer. As mentioned previously, the nominal thicknesses of the three PE sheets (from left to right) are 100, 35 and 230 µm; the corresponding measured thicknesses are 139, 39 and 241 µm, respectively. While these micrometer measurements are all roughly 10% higher than the fitted values, they do not account for the 30% discrepancies on the left. For a better illustration, the transmission ratios calculated using these PE thicknesses are shown in Figs. 11 ▸(*a*). Consequently, a plausible explanation for the leftmost discrepancy is that the supplier inadvertently sent a PE sheet with a nominal thickness of 125 or 130 µm instead of the ordered specification. Notably, a pristine PE sheet with a nominal thickness of 230 µm (the same thickness as in the IMAT measurements) was accurately determined with the RADEN measurements. This result strongly supports our previous hypothesis regarding a stoichiometric deviation in the neutron-irradiated PE that was measured on IMAT (see Appendix *A*6).

In regions without PE sheets, the fitted PE thicknesses are close to zero, maintaining an uncertainty within 4 µm, which corresponds to 11 wt p.p.m. hydrogen in PE. Given these overall fitting uncertainties and the background values obtained in the bare regions, the methodology developed in this work successfully resolves a hydrogen detection limit down to the 10 wt p.p.m. level.

In this work, quantifying additional hydrogen signals relies heavily on a reference sample or region. For the RADEN measurements, we utilized the exact same SDSS sample for reference. This setup, combined with a precise cross-section model and well controlled experimental instrumentation, enables us to push the hydrogen detection limit down to the 10 wt p.p.m. level. A reference sample is critical when trying to detect such a weak signal, which represents less than a 0.5% change. At this level, omitting the reference sample would compromise the validity of the method; for instance, any inhomogeneity in sample thickness or composition would cause variations in transmission intensity, making it difficult to distinguish whether the signal change is driven by hydrogen or variations in other physical quantities. While this method remains applicable to the IMAT measurements, where we lacked a strict reference and instead used a ‘similar’ SDSS region (in terms of thickness and element composition), the significant contrast (100 wt p.p.m. hydrogen variation) and the homogeneity between these regions made detection feasible.

## 6. Conclusions

Motivated by the need for quantitative detection of very small hydrogen signals in metallic materials, this work establishes a universal and comprehensive analysis workflow for wavelength-resolved neutron transmission imaging. The workflow is built entirely on open-source components – most critically the *NCrystal* toolkit – and is designed to enable reliable quantification even when hydrogen-equivalent concentrations are far below levels accessible with conventional imaging.

A series of key contributions have been demonstrated:

(i) A systematic simulation study was performed to quantify forward-scattering artefacts under different sample-to-detector distances. By benchmarking *OpenMC*+*NCrystal* against the established work by Sato *et al.* (2017 ▸) and extending the simulations to a wide range of materials and experimental slit configurations, we provide practical guidance on when forward scattering can be safely neglected and when it becomes significant and requires correction. A few examples for different materials are provided in Appendix *A*[App appa] to offer interested readers practical guidance when planning experiments.

(ii) The developed least-squares fitting routine – based on *NCrystal* cross sections – accurately reproduced the measured spectra after applying the normalization and correction procedures described in this work. The model effectively incorporated the known chemical compositions while adjusting microstructural parameters such as extinction and lattice constants.

(iii) Using Super Duplex stainless steel with polyethylene attachments as a controlled proxy for low hydrogen-equivalent content, we have demonstrated that the workflow can reliably determine polyethylene thicknesses with micrometre-level uncertainty, validating the hydrogen detection limit down to 10 wt p.p.m. needed for low-level hydrogen quantification. The same workflow is readily applicable to quantifying other elements and is expected to be highly useful for a wide variety of time-of-flight neutron imaging experiments.

(iv) All analysis tools, including the fitting script, have been made publicly available to support reproducibility and to facilitate the wider adoption of this workflow within the neutron imaging community.

(v) More generally, this work highlights that the dynamic state of atoms contributes directly to wavelength-resolved neutron transmission and therefore represents an additional contrast mechanism, namely spectroscopic contrast. While this must be accounted for during quantitative analysis, it also opens new opportunities, *e.g.* to distinguish different hydrogen trapping states and local atomic environments using neutron imaging.

Overall, the results highlight that well characterized reference states are essential for absolute quantification, particularly when targeting trace hydrogen concentrations. In this context, *in situ* experiments, where the hydrogen-free initial state of the material serves as an internal reference, are essential for achieving accurate quantitative analysis.

The comparison between the IMAT and RADEN measurements further indicates that detector-related systematic effects can introduce wavelength-dependent offsets in normalized transmission spectra. Although the exact origin of the detector-generated background could not be identified conclusively, the results demonstrate that such systematic effects should be considered in highly quantitative wavelength-resolved neutron imaging, and that the implemented OA-based correction effectively compensates for the resulting wavelength-dependent offset.

More broadly, this study demonstrates that the combination of rigorous Monte Carlo simulations as a digital twin, physically grounded cross-section modelling and well controlled experimental instrumentation enables quantitative neutron imaging at pulsed neutron sources. In the future, more detailed beamline models, such as those implemented in Monte Carlo codes like *McStas* or *VITESS*, could be incorporated to improve further the accuracy of the simulations beyond the simplified beam geometry used here. The workflow presented here establishes a practical and transferable approach for routinely detecting and quantifying weak transmission signals across a wide range of materials and applications. In this context, the quantitative detection of low-level hydrogen down to 10 wt p.p.m. (modelled by polyethylene in this work) in Fe-based engineering alloys, central to the study of hydrogen embrittlement, represents a particularly important application.

## Figures and Tables

**Figure 13:**
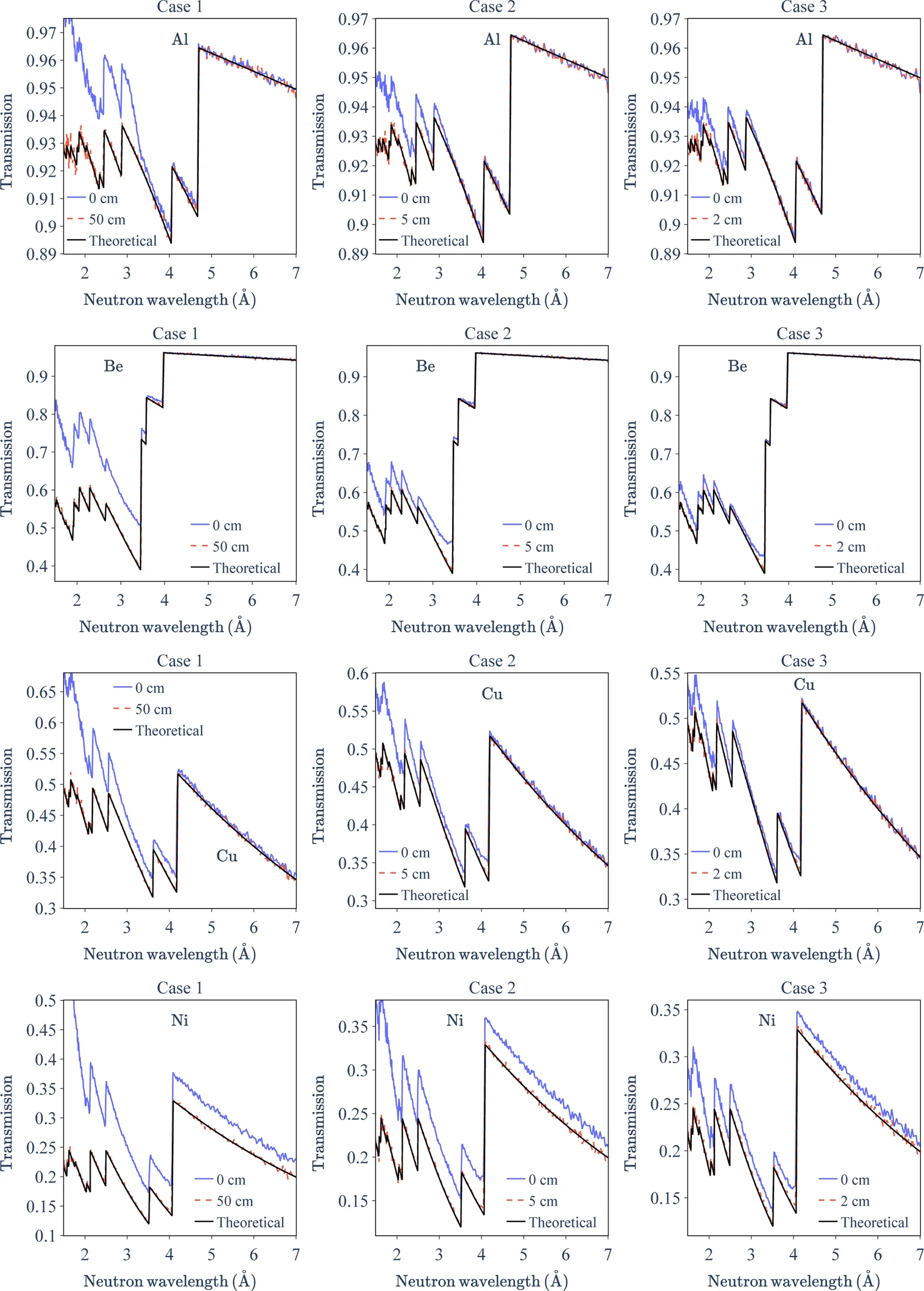
Comparison of simulated and theoretically predicted transmission spectra (solid black lines) for 8 mm thick Al, Be, Cu and Ni across simulation Cases 1, 2 and 3. The results indicate that the sample-to-detector distances of 50 cm, 5 cm and 2 cm are sufficient to mitigate forward-scattering artefacts in Cases 1, 2 and 3, respectively.

**Figure 14:**
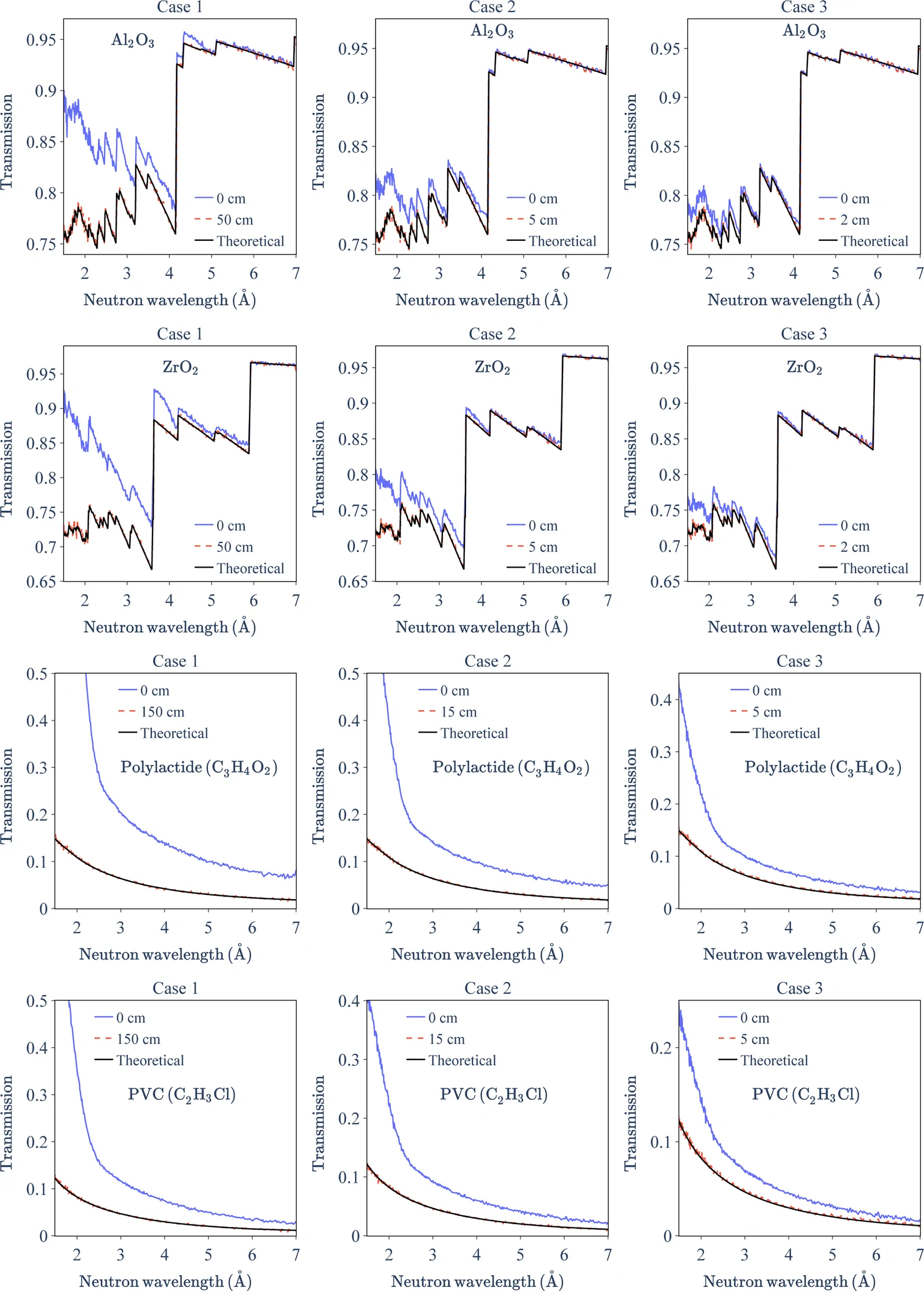
Comparison of simulated and theoretically predicted transmission spectra (solid black lines) for 8 mm thick Al_2_O_3_, ZrO_2_, polylactide (C_3_H_4_O_2_) and polyvinylchloride (PVC; C_2_H_3_Cl) across simulation Cases 1, 2 and 3. The results indicate that the sample-to-detector distances of 50 cm, 5 cm and 2 cm are sufficient to mitigate forward-scattering artefacts in Cases 1, 2 and 3 for Al_2_O_3_ and ZrO_2_. However, polylactide and PVC necessitate larger sample-to-detector distances of 150 cm, 15 cm and 5 cm for the three cases, respectively.

**Figure 15:**
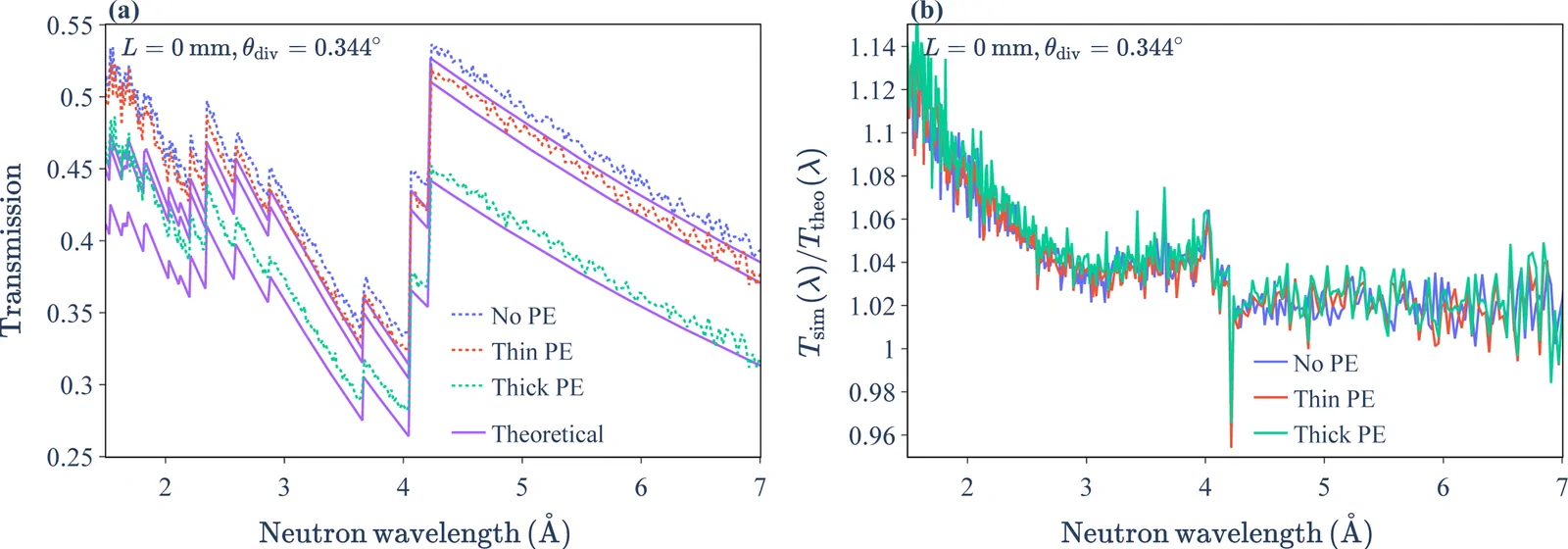
(*a*) Forward-scattering artefacts at zero sample-to-detector distance for three different ROIs. (*b*) Ratios of simulated to theoretical transmission spectra, highlighting that the forward-scattering effects remain consistent across these ROIs. The drops in the data near the Bragg cutoff (4.2 Å) are an artefact of the binning in the simulated transmission spectra.

**Figure 16:**
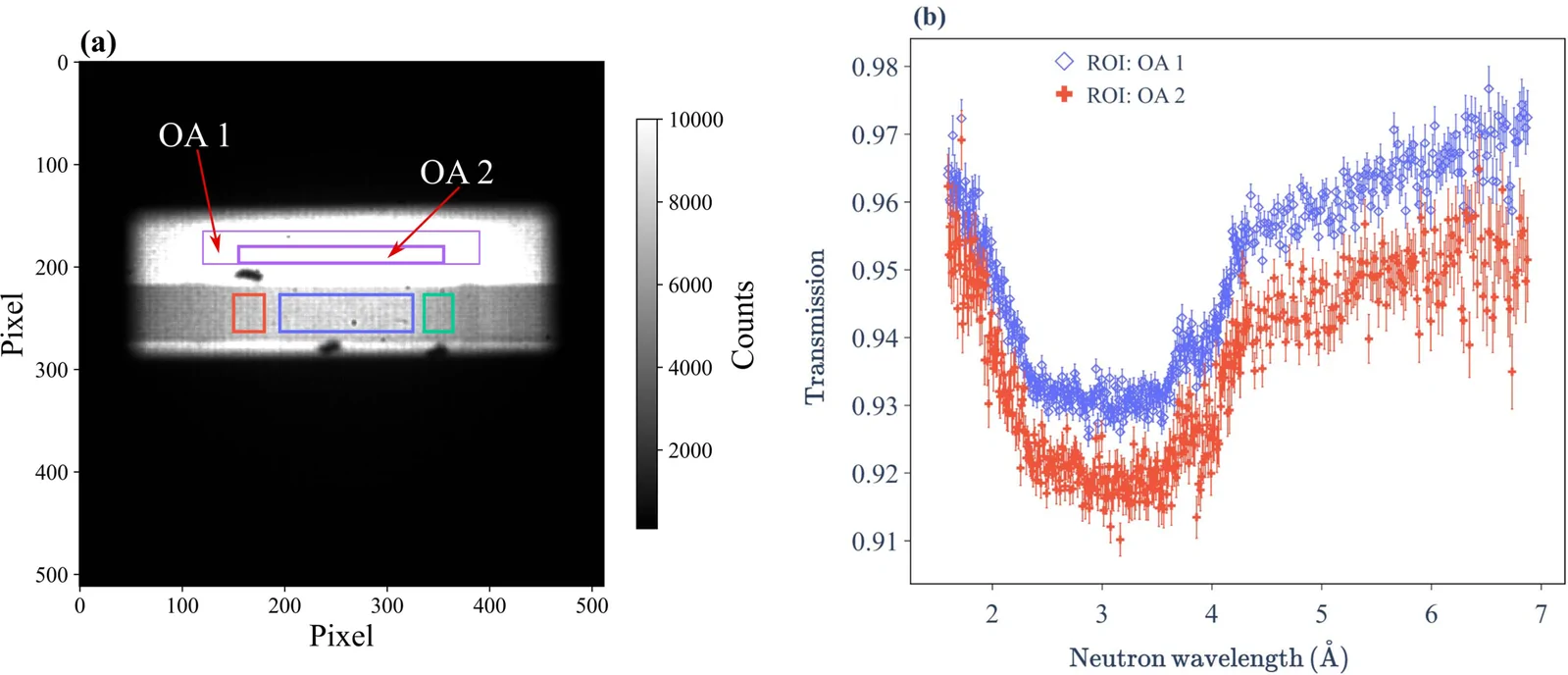
(*a*) Radiograph measured for the front configurations measured on IMAT. Two ROIs within the OA regions are indicated, denoted OA1 and OA2. (*b*) Corresponding wavelength-resolved neutron transmission spectra for OA1 and OA2. The spectrum of OA1 is identical to that shown in the upper plot of Fig. 6(*c*).

**Figure 18:**
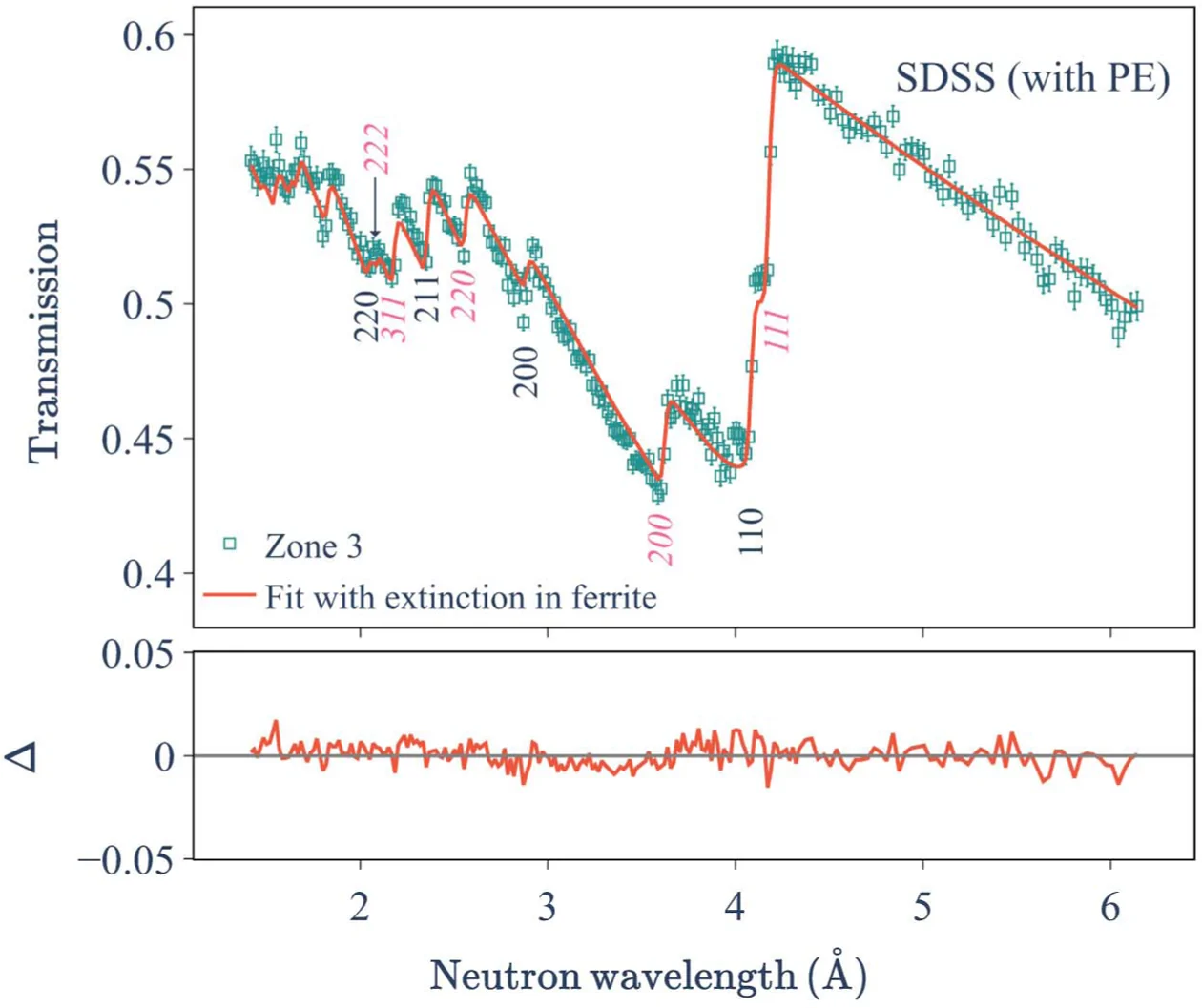
*NCrystal*-based least-squares fit of the SDSS sample with PE covers, using transmission spectrum data from Zone 3 [indicated in the middle panel of Fig. 7(*c*)].

**Table 1:** Nominal element compositions (in wt%) of the Fe-based SDSS (AZo Materials, 2025 ▸) The term ‘balance’ refers to the remaining Fe content that makes up the SDSS sample.

Grade	C	Si	Mn	Cr	Ni	Mo	N	Fe
SDSS	≤ 0.03	≤ 0.8	≤ 1.2	25	7	4	0.3	Balance

**Table 2:** Results of the parameter fit for the SDSS grade without PE attachments, showing the free parameters, their values and the associated correlation matrix For clarity, only the lower triangle of the symmetric matrix is displayed.

Parameter	Fitted value	Correlation
Scale factor *A*	0.993 (1)	1
Ferrite phase fraction	0.49 (2)	0.12 1
Ferrite crystallite size 2*r* (µm)	1.20 (8)	−0.00, −0.62, 1
Ferrite mosaic spread parameter, *g*_α_ (rad^−1^)	1.3 (1) × 10^3^	−0.51, 0.38, −0.67, 1
Austenite mosaic spread parameter, *g*_γ_ (rad^−1^)	9 (2) × 10	−0.03, 0.75, −0.71, 0.31, 1

## Data Availability

The data supporting the results reported can be accessed upon request.

## Appendix A

### A1. Validation of *OpenMC*+*NCrystal* simulations

Our *OpenMC*+*NCrystal* simulations replicated the configuration shown by Sato *et al.* (2017 ▸), thereby modelling a pulsed neutron source of 10 × 10 cm with zero divergence incident on a b.c.c. iron plate, with a TOF detector positioned a distance *L* downstream. A schematic presentation of the configuration is illustrated in Fig. 2 ▸(*a*). To allow for a direct comparison with the results shown by Sato *et al.* (2017 ▸), a first simulation was performed for a 6 mm thick plate using 2 × 10^10^ neutrons distributed uniformly between 1 and 6 Å, ensuring sufficient statistics for the transmission spectrum calculations. The detector distance was varied from 0 to 150 cm from the sample, while the ROI was maintained at 10 × 10 cm. The resulting transmission spectra are presented in Fig. 12 ▸(*a*). The theoretically predicted transmission spectrum is shown as a solid black line. Transmission signal artefacts increase as the sample-to-detector distance decreases. This effect is most pronounced at neutron wavelengths below the 110 Bragg edge, confirming that the increased transmission signal is primarily due to the detection of forward Bragg-scattered neutrons at small sample-to-detector distances. Our simulation results are in good agreement with Fig. 2 of Sato *et al.* (2017 ▸), thus verifying the capability of *OpenMC*+*NCrystal* in modelling transmission experiments.

A second simulation was performed for an 18 mm thick plate using the same configuration. The resulting transmission spectra are compared with the theoretical predictions in Fig. 12 ▸(*b*). As expected, the transmission intensities are lower than those for the 6 mm plate due to increased attenuation. Both simulated spectra exhibit a similar trend of decreasing forward-scattering artefacts with increasing sample-to-detector distance. Additionally, both simulations confirm that the impact of forward scattering can be neglected only at distances greater than 50 cm for this specific configuration.

We investigated the influence of detection area and position on the transmission spectra to compare our results with those presented by Sato *et al.* (2017 ▸). A third simulation was conducted, identical to the first but employing 2 × 10^11^ neutrons to improve statistics for the smaller pixel-sized ROIs (0.8 × 0.8 mm). The resulting spectra for a pixel located at the centre and one at the corner (4.2 cm, 4.2 cm) are shown in Figs. 12 ▸(*c*) and 12 ▸(*d*), respectively. These results are in very good agreement with Figs. 5 and 6 in Sato *et al.* (2017 ▸). The central pixel displays more pronounced forward-scattering artefacts compared with the corner pixel.

By employing the same configurations as the reference study, our *OpenMC*+*NCrystal* simulation results align closely with those reported by Sato *et al.* (2017 ▸), validating our simulation framework.

### A2. Simulations for more materials in different sample-to-detector distances

Having established a comprehensive *OpenMC*+*NCrystal* simulation toolkit in this work, we broadened the scope of the study by performing simulations across several commonly investigated materials, making use of the extensive *NCrystal* database of crystalline and amorphous solids, liquids and gases. This broader survey aims to compare how strongly forward-scattering artefacts manifest across different classes of materials. Additional results are presented in Figs. 13 ▸ and 14 ▸, which may serve as practical reference cases for researchers designing quantitative neutron transmission experiments for different material systems.

### A3. Discussion regarding the forward-scattering effects across different ROIs

As shown in Fig. 4 ▸, Monte Carlo simulations prove that the effects of forward scattering can be safely neglected for the selected ROIs (corresponding to no PE, thin PE and thick PE, respectively) in the IMAT experimental setup at a sample-to-detector distance of 20 mm. Figs. 15 ▸(*a*) and 15 ▸(*b*) illustrate these effects at zero sample-to-detector distance. Specifically, Fig. 15 ▸(*a*) highlights the forward-scattering artefacts for the three ROIs, while Fig. 15 ▸(*b*) displays the ratio between the simulated transmission *T*_sim_(λ) and the theoretical transmission *T*_theo_(λ). The ratios demonstrate that the effects are highly similar across these ROIs; because the attached PE sheets are relatively thin (≤ 0.25 mm), any additional scattering effects remain negligible.

### A4. Discussion regarding the OA correction

In this section, we discuss how the choice of ROI within the OA region impacts the corrected transmission intensity. First, we calculated the wavelength-resolved transmission spectra for two specific ROIs, denoted OA1 and OA2 in Fig. 16 ▸(*a*) [where OA1 is identical to the ROI in Fig. 6 ▸(*a*)]. The corresponding neutron transmission spectra are shown in Fig. 16 ▸(*b*).

The transmission spectra for both OA regions exhibit a wavelength dependency. Additionally, the transmission intensity of OA1 is consistently higher than that of OA2. By dividing the transmission spectrum of each SDSS ROI by the wavelength-dependent intensity of OA1, we obtained the OA-corrected transmission spectra, as illustrated in Fig. 6 ▸(*c*). As shown in Fig. 6 ▸(*d*), using OA1 to evaluate the transmission intensity yields a consistent ratio of unity between the front and back transmission.

Conversely, if OA2 is used for the correction, a consistent ratio of 1.01 is observed between the transmission spectra of the front and back configurations. While this minor deviation does not impact the final PE thickness fits, as it cancels out when dividing the transmission of the no-PE region by that of the PE-attached region, this observation underscores the necessity of using a reference region on the same sample as a baseline. This internal baseline ensures that systematic effects cancel out, thereby enhancing the overall robustness of the methodology and preventing minor experimental variations from compromising the accuracy of the analysis.

Such precision is particularly critical when quantifying signals from weak contributions, where precise baseline subtraction is required to isolate subtle spectral variations. It should be noted that, if left unaccounted for, a 1% deviation in the transmission signal would correspond to approximately 30 wt p.p.m. of hydrogen in the current sample configuration. When aiming to detect such small signal changes, ruling out systematic errors from the experimental setup is of the utmost importance. The observed OA transmission below unity cannot be explained by scattering from the sample into the OA, as such scattering would instead increase the detected intensity. Instead, the observations are consistent with detector-related parasitic scattering. A plausible explanation is that the detector-generated background (generated inside the detector) differs between the open-beam and sample measurements because the presence of the sample modifies the neutron field experienced by the detector system. Consequently, normalization using the open-beam measurement produces a wavelength-dependent offset in the apparent transmission. This interpretation is further supported by the repeat measurements performed on the RADEN beamline using a different detector configuration, where the OA transmission remained much closer to unity over the full wavelength range [top panel of Fig. 7 ▸(*c*)]. While the exact origin of the detector-related background cannot be identified conclusively from the present measurements, the comparison indicates that the effect is associated with the detector configuration rather than with changes in the incident neutron spectrum.

### A5. Parameter determination of Jorgensen resolution function for IMAT

According to Von Dreele *et al.* (1982 ▸), the resolution can be calculated using

where *C* is a characteristic constant of the instrument. By parameterizing α = α_1_/*d*, β = β_0_ + β_1_/*d*^4^ and Σ = Σ_1_*d* (where *d* is the interplanar spacing), the resolution function can be written as

Additionally, because the parameter β represents the decay process in the Jorgensen model, a corresponding decay time  can be defined as

where *C*′ is a scaling constant.

The decay time and resolution functions were fitted simultaneously against the wavelength-dependent experimental data digitized from Fig. 6 of Ramadhan *et al.* (2019 ▸). During the nonlinear least-squares refinement using *lmfit*, the parameters (Σ_1_ = 1.43 × 10^−3^, α_1_ = 100000 and *C*′ = 20000) were kept fixed. Specifically, Σ_1_ was predetermined from the ratio of the moderator thickness (8 cm) of ISIS Target Station 2 and the flight path length (56.1 m) of IMAT. The values of α_1_ and *C*′ were fixed, because allowing them to vary freely did not significantly improve the quality of the fit, but instead introduced large uncertainties in the remaining parameters.

As shown in Fig. 17 ▸, the fitted curves are in good agreement with the experimental data, yielding β_0_ = 45 (2), β_1_ = 58 (6) and *C* = 0.0140 (5). Consequently, these refined parameters for the Jorgensen-type resolution function of the IMAT instrument can be fixed for transmission spectrum fittings for the SDSS data (Section 5.2.1). This fitting approach for the Jorgensen-type resolution function can be readily applied to other pulsed neutron beamlines, such as RADEN.

### A6. Discussion regarding the H/C ratios for the thick PE sheet measured on IMAT

The 10% discrepancy of the fitted thickness for the thick PE sample may stem from a reduced hydrogen-to-carbon (H/C) ratio, dropping from the stoichiometric value of 2 to approximately 1.8. This reduction is likely to be caused by radiation-induced material modifications, as this particular sample had previously been exposed to neutrons for extended periods at the ISIS facility.

Under neutron irradiation, the actual H/C ratio can fall below 2 if the material undergoes dehydrogenation: radiation creates radicals, some H is abstracted, H_2_ can evolve and unsaturated groups such as C=C can form. In irradiated PE, the reported chemistry includes crosslinking, chain scission, oxidation and formation of double bonds, with explicit evidence that some hydrogen atoms important for neutron shielding are released. Neutron interactions in hydrocarbon polymers are also often treated as producing recoil protons, so the radiation chemistry resembles proton irradiation (O’Donnell, 1989 ▸). For polymer neutron shields in storage/transport systems, the literature notes that, in many practical cases, thermal ageing and oxidation dominate over irradiation as degradation mechanisms (Wolff *et al.*, 2019 ▸). Notably, the pristine PE sheets used in the RADEN measurements do not show this effect (see Section 5.2.2).

### A7. Fitting the PE thickness without using a reference

In practice, the PE thickness can be fitted simultaneously with all other free parameters for the PE-covered regions. As an example, Fig. 18 ▸ shows the fitting results of the transmission spectrum of Zone 3 measured on RADEN [shown in the middle panel of Fig. 7 ▸(*c*)].

The fitting transmission shows good agreement with the experimental data, yielding a reduced χ^2^ value of 2.4. The directly fitted PE thickness is 68 (7) µm, which is nearly twice the nominal value (35 µm). This discrepancy is primarily attributed to a strong correlation (0.97) between the PE thickness and the scale factor; in contrast, the correlation between the thickness and all other free parameters remains below 0.25. Consequently, any systematic error in the scale factor directly propagates into the estimated PE thickness. This highlights the importance of utilizing a reference sample, as dividing the transmission spectra minimizes the impact of the scale factor. Alternatively, one can determine the scale factor and other free parameters from the reference sample and fix them when fitting the PE thickness from the PE-covered region. Although this indirect approach fits the spectrum directly rather than the ratio, it is mathematically equivalent to fitting the spectral ratio shown in Figs. 10 ▸ and 11 ▸.
